# Supplementary material for: Dividing Cells Regulate Their Lipid Composition and Localization
Source: Cell. 2014 Jan 30;156(3):428–39. doi: 10.1016/j.cell.2013.12.015 (PMC3909459; doi:10.1016/j.cell.2013.12.015)
Supplement: Document S1. Tables S1 and S3 [file mmc1.pdf]

Table S1. Mass-to-Charge Ratios (m/z) and LC Retention Times (RT) of Lipids that Change during Cytokinesis and at the Midbody, Related to Table 1
For chemical structures of the lipids, see Fig. 1.

m/z	RT (min)	Lipid	Fold increase during cytokinesis [(a)]	Fold increase at midbody [(b)]
441.3344	~40	sterol derivative	>27	unchanged
661.5191	~46	phosphatidic acid ether/ester (O-18:0/16:0)	40	unchanged
837.5525	~54	phosphatidylinositol (16:0/18:0)	10	unchanged
538.5216	~70	C16 diH-ceramide	>11	unchanged
566.5518	~72	C18 diH-ceramide	accumulated[(c)]	unchanged
594.5830	~73	C20 diH-ceramide	accumulated[(c)]	unchanged
622.6170	~75	C22 diH-ceramide	accumulated[(c)]	accumulated[(c)]
650.6456	~77	C24 diH-ceramide	accumulated[(c)]	accumulated[(c)]
620.5987	~77	C22 ceramide	4	4
648.6366	~79	C24 ceramide	4	14
698.5610	~68	C16 hexosylceramide	>16	13
810.6783	~79	C24 hexosylceramide	unchanged	>36
647.4645	~48	phosphatidic acid (16:0/16:0)	unchanged	14
792.7113	~72	triacylglycerol (16:1,12:0, 18:1)	unchanged	54
810.5278	~49	phosphatidylserine (18:0/20:4)	unchanged	6

[(a)] Fold increase in S-phase vs cytokinesis cells is determined by [Abundance $_{\text{cytokinesis}}$] / [Abundance $_{\text{S-phase}}$] for each lipid. Abundance is the total ion count for a given ion. Each ion corresponds to a mass-to-charge ratio (m/z), which is used to assign the lipid species.
[(b)] Fold increase in midbody vs cytokinesis cells is determined by [Abundance $_{\text{midbody}}$] / [Abundance $_{\text{purified lysate}}$] for each lipid.
[(c)] A numeric value for fold increase could not be calculated due to the low abundance of these ions in S-phase or cytokinesis cells.

Table S3. Targeted Analysis of the Sphingolipids in siSMPD4-, siGALC-, and siDGAT2-Treated Cells, Related to Figure 3

Fold changes in ceramides, diH-ceramides, hexosylceramides and sphingomyelins were calculated in siRNA treated cells. Fold changes are an average of two independent experiments. For a graphical representation see Fig. S3A.

		siSMPD4	siGALC	siDGAT2
		Average fold changes relative to control		
CERAMIDES	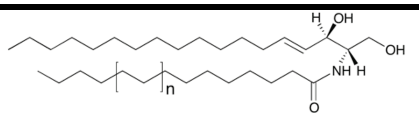			
	n=1 (C14 ceramide)	not detected	not detected	not detected
	n=2 (C16 ceramide)	4.5x increase	unchanged	unchanged
	n=3 C18 ceramide)	not detected	not detected	not detected
	n=4 (C20 ceramide)	not detected	not detected	not detected
	n=5 (C22 ceramide)	8x increase	unchanged	unchanged
	n=6 (C24 ceramide)	3.5x increase	unchanged	unchanged
diH-CERAMIDES	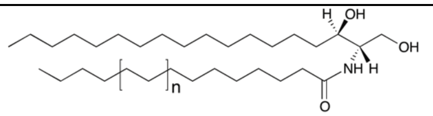			
	n=1 (C14 diH-ceramide)	not detected	not detected	not detected
	n=2 (C16 diH-ceramide)	not detected	not detected	not detected
	n=3 (C18 diH-ceramide)	not detected	not detected	not detected
	n=4 (C20 diH-ceramide)	not detected	not detected	not detected
	n=5 (C22 diH-ceramide)	not detected	not detected	not detected
	n=6 (C24 diH-ceramide)	not detected	not detected	not detected
HEXOSYLCERAMIDES	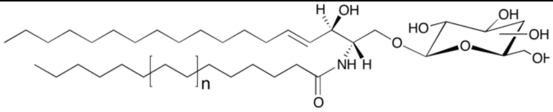			
	n=1 (C14 hexosylceramide)	unchanged	unchanged	unchanged
	n=2 (C16 hexosylceramide)	3.1x increase	3.2x increase	unchanged
	n=3 (C18 hexosylceramide)	unchanged	2.1x increase	3.5x increase
	n=4 (C20 hexosylceramide)	unchanged	unchanged	unchanged
	n=5 (C22 hexosylceramide)	unchanged	unchanged	unchanged
	n=6 (C24 hexosylceramide)	2.2x increase	2.1x increase	unchanged
SPHINGOMYELINS	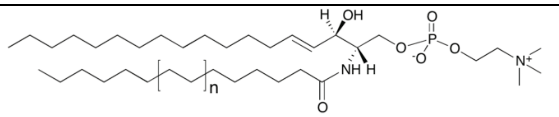			
	n=1 (C14 SPM)	unchanged	unchanged	unchanged
	n=2 (C16 SPM)	unchanged	unchanged	unchanged
	n=3 (C18 SPM)	unchanged	unchanged	unchanged
	n=4 (C20 SPM)	not detected	not detected	not detected
	n=5 (C22 SPM)	unchanged	unchanged	unchanged
	n=6 (C24 SPM)	unchanged	unchanged	unchanged
