# Supplementary material for: Dividing Cells Regulate Their Lipid Composition and Localization
Source: Cell. 2014 Jan 30;156(3):428–39. doi: 10.1016/j.cell.2013.12.015 (PMC3909459; doi:10.1016/j.cell.2013.12.015)
Supplement: Document S2. Article plus Supplemental Information [file mmc7.pdf]

Cell

Dividing Cells Regulate Their Lipid Composition and Localization

G. Ekin Atilla-Gokcumen,[1,6,7] Eleonora Muro,[2,6] Josep Relat-Goberna,[3] Sofia Sasse,[1,4] Anne Bedigian,[1] Margaret L. Coughlin,[5] Sergi Garcia-Manyes,[3] and Ulrike S. Eggert[1,2,*]
[1]Department of Biological Chemistry and Molecular Pharmacology and Dana-Farber Cancer Institute, Harvard Medical School, Boston, MA 02115, USA
[2]Department of Chemistry and Randall Division of Cell and Molecular Biophysics, King's College London, London SE1 1UL, UK
[3]Department of Physics and Randall Division of Cell and Molecular Biophysics, King's College London, London WC2R 2LS, UK
[4]Institute for Neurobiology, Westfälische Wilhelms-Universität Münster, 48149 Münster, Germany
[5]Department of Systems Biology, Harvard Medical School, Boston, MA 02115, USA
[6]These authors contributed equally to this work
[7]Present address: Department of Chemistry, The State University of New York, Buffalo, NY 14260, USA
*Correspondence: ulrike.eggert@kcl.ac.uk
http://dx.doi.org/10.1016/j.cell.2013.12.015
This is an open-access article distributed under the terms of the Creative Commons Attribution License, which permits unrestricted use, distribution, and reproduction in any medium, provided the original author and source are credited.

SUMMARY

Although massive membrane rearrangements occur during cell division, little is known about specific roles that lipids might play in this process. We report that the lipidome changes with the cell cycle. LC-MS-based lipid profiling shows that 11 lipids with specific chemical structures accumulate in dividing cells. Using AFM, we demonstrate differences in the mechanical properties of live dividing cells and their isolated lipids relative to nondividing cells. In parallel, systematic RNAi knockdown of lipid biosynthetic enzymes identified enzymes required for division, which highly correlated with lipids accumulated in dividing cells. We show that cells specifically regulate the localization of lipids to midbodies, membrane-based structures where cleavage occurs. We conclude that cells actively regulate and modulate their lipid composition and localization during division, with both signaling and structural roles likely. This work has broader implications for the active and sustained participation of lipids in basic biology.

INTRODUCTION

As a cell divides, it undergoes massive shape changes, culminating in the formation of a small structure, the midbody, where cleavage between daughter cells occurs (Eggert et al., 2006b). Traditionally, cell division research has focused primarily on the actin and microtubule cytoskeletons as determinants of cell shape and as drivers of cell division. Although membranes are intimately connected to the cytoskeleton and intact membranes are an absolute requirement after division has been completed, little is known about the role of membranes during cell division. For example, starting at the most basic level, we do not know whether cells change their lipid composition as they go through the cell cycle. We report here a comprehensive and systematic analysis of changes in the lipid composition and localization during cell division.

Membranes and membrane trafficking are important to stabilize changes in curvature and to provide membrane to alleviate tension caused by shape changes during division. Membranes are also essential in signaling and are involved in the transport and modulation of key proteins at constriction and scission sites (Albertson et al., 2005; Echard, 2008; Eggert et al., 2004; Montagnac et al., 2008; Zhang et al., 2012). A recent report showed that, in dividing sea urchin eggs, new membrane addition is also required at the poles (Gudejko et al., 2012). Membrane lipids can achieve these different outcomes through different interactions with their binding partners, which are mostly proteins and/or other lipids. For example, some lipid species might interact with proteins to form local signaling platforms in vesicles or at the plasma membrane, whereas other lipid species might provide mechanical support for membrane architectures such as curved structures (van Meer et al., 2008). Local transport and/or synthesis and turnover of different lipid species are likely to be essential in regulating their varied functions. Except for a few examples of well-studied lipids, we are only beginning to appreciate the critical and diverse roles that lipids play during many biological processes.

Cells express hundreds of enzymes that synthesize lipids and produce tens of thousands of different lipids. Except for a few specific cases, it is unclear why cells invest energy into producing such complex and diverse lipidomes. Compared to other biological macromolecules, we have a poor understanding of the roles of specific lipids in biological processes. One of the reasons why it has been difficult to study lipids in their biological context is that, compared to proteins, there are fewer standard methods to visualize and manipulate lipid levels and localizations in cells (Saario et al., 2012; Schultz, 2010). There have been hints that a few different lipids might be involved in cell division (Atilla-Gokcumen et al., 2011; reviewed in Atilla-Gokcumen et al., 2010)

mostly by showing that fluorescent markers for these lipids localize to cytokinesis structures (Emoto et al., 2005; Ng et al., 2005). Only phosphatidylinositol 4,5-bisphosphate (PIP2) (Field et al., 2005) and the related phosphatidylinositol 3,4,5-triphosphate (PIP3) (Sagona et al., 2010), well-known signaling lipids that regulate actin polymerization and membrane trafficking (Echard, 2012), have been studied extensively. Here, we use mass spectrometry to identify which lipid species change as cells divide and dissect localized contributions at the midbody. We systematically perturb lipid levels in cells by knocking down lipid biosynthetic enzymes and use atomic force microscopy (AFM) to analyze biophysical properties of dividing and perturbed cells. Having determined the lipid complement of dividing cells, our combined approach is now allowing us to form hypotheses about the biological roles of the lipids that we identified.

RESULTS

A Lipidomic Comparison of Dividing and Nondividing Cells Reveals Cell-Cycle-Dependent Lipid Composition

We used liquid chromatography-mass spectrometry (LC-MS)-based global lipid profiling, an unbiased approach that does not require lipid perturbations (Saghatelian et al., 2004), to analyze the lipidome of cells synchronized at different stages of the cell cycle. Lipids are a broad class of metabolites that vary in structure and size. Based on this diversity, different ionization techniques can be used to analyze different classes and subgroups of lipids. For lipidomic studies, the use of ionization methods that do not cause extensive fragmentation is preferred because it enables the detection of a wide range of lipids within complex mixtures. We used electrospray ionization (ESI) (Han and Gross, 2003), which allows the analysis of different lipids from total lipid extracts over a range of mass-to-charge (m/z) ratios. We compared total lipids extracted from HeLa cells at cytokinesis to cells at S phase or the metaphase stage of mitosis and confirmed the identity of predicted lipid species by tandem MS (Table 1, Figure 1, and Table S1 available online). The lipid composition of cells in mitosis was similar to cells in cytokinesis. The timing of division relative to other stages of the cell cycle is rapid, suggesting that there may not be enough time for the global synthesis of new lipids, similar to protein translation not playing a major role during cell division (Stumpf et al., 2013).

The lipid composition of cells in S phase differs from cells in cytokinesis. Specifically, 11 lipid species out of the many thousand species synthesized (and detected by LC-MS) accumulate at least 4-fold in dividing (measured in cytokinesis but unchanged in mitosis) cells (Table 1 and Figure 1). Remarkably, all of these lipids are very specific species within different families. This specificity is exciting because it shows that cells are highly precise in their lipid regulation, but it also presents analytical challenges because tools are barely available to study lipid families, let alone specific species within these families. Biological roles have not been reported for two species that we identified: an unusual sterol derivative and an ether/ester-linked phosphatidic acid (rather than a traditional diester). The geometry and hydrophobic profile of the sterol derivative (hydroxy cholestane) is different from traditional sterols, such as cholesterol, due to the absence of a double bond in the B ring and the additional hydroxyl groups. Ether-linked phospholipids are rare structures (Ivanova et al., 2010). Their biosynthesis varies significantly from traditional glycerolipids, and it involves the esterification of acetyl CoA as the first step. These lipids, with a more lipophilic head group due to the ether linkage, can cause changes in the arrangement of lipids within membranes (Braverman and Moser, 2012). The upregulation of both of these lipid species during cell division suggests previously undescribed functions.

Eight of the lipids that we identified are specific subspecies within the sphingolipid family. Sphingolipids are a large lipid family based around a sphingosine base. They often include a fatty acid (then called ceramide) and can be highly glycosylated. Sphingolipids have been implicated in several biological processes, including regulation of apoptosis, inflammatory response, autophagy, and motility (Hannun and Obeid, 2011; Meivar-Levy et al., 1997). The first clues for a role for ceramides

Table 1. Specific Lipids Accumulate in Dividing Cells and at Midbodies

LIPID	Fold Increase during Cytokinesis[a]	Fold Increase at the Midbody[b]
Sterol derivative	>27	unchanged
Phosphatidic acid ether/ester (O-18:0/16:0)	40	unchanged
Phosphatidylinositol (16:0/18:0)	10	unchanged
C16 diH-ceramide	>11	unchanged
C18 diH-ceramide	accumulated[c]	unchanged
C20 diH-ceramide	accumulated[c]	unchanged
C22 diH-ceramide	accumulated[c]	accumulated[c]
C24 diH-ceramide	accumulated[c]	accumulated[c]
C22 ceramide	4	4
C24 ceramide	4	14
C16 hexosylceramide	>16	13
C24 hexosylceramide	unchanged	>36
Phosphatidic acid (16:0/16:0)	unchanged	14
Triacylglycerol (16:1, 12:0, 18:1)	unchanged	54
Phosphatidylserine (18:0/20:4)	unchanged	6

Results of LC-MS lipidomic analysis of S phase versus cytokinesis cells (middle column) and midbodies versus mock midbody purification from asynchronous lysate (right column). Lipids and their corresponding fold increases are shown (averages of three independent profiling experiments). The chemical structures of these lipids are shown in Figure 1. See Table S1 and Figures 5D and S1 for additional details.

[a]Fold increase in S phase versus cytokinesis cells is determined by [$Abundance_{cytokinesis}$] / [$Abundance_{S\text{-}phase}$] for each lipid. Abundance is the total ion count for a given ion. Each ion corresponds to a mass-to-charge ratio (m/z), which is used to assign the lipid species.

[b]Fold increase in midbody versus cytokinesis cells is determined by [$Abundance_{midbody}$] / [$Abundance_{purified\ lysate}$] for each lipid.

[c]A numeric value for fold increase could not be calculated due to the low abundance of these species in S phase or cytokinesis cells.

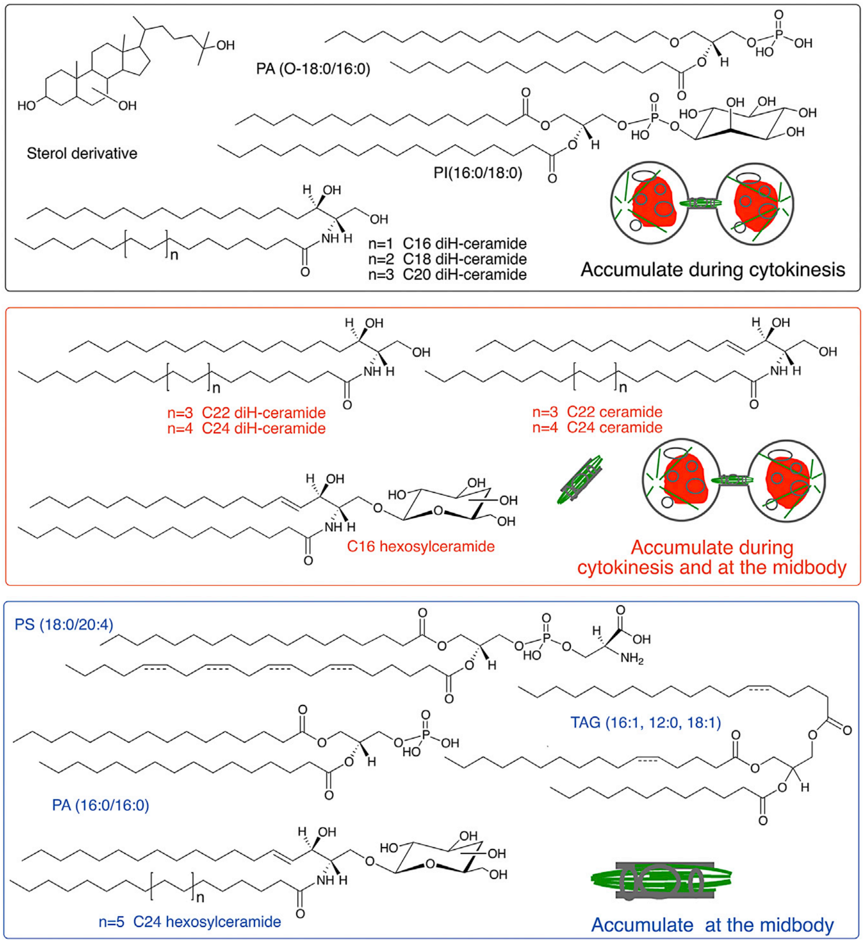

Figure 1. Chemical Structures of Lipids that Change during Cytokinesis and at the Midbody

Chemical structures of the lipids listed in Table 1 are shown. Lipids that accumulate from S phase to cytokinesis are shown in black and red. Lipids that accumulate at the midbody are shown in red and blue. Note that the *sn*-1 and *sn*-3 positions and the positions of the double bonds in TAG (16:1, 12:0, 18:1) and PS (18:0/20:4) and the position of the third hydroxyl group on the B ring of the tri-hydroxycholestane could not be determined. Targeted MS analysis of the ceramide and diH-ceramide content of cytokinesis cells and mid-bodies is shown in Figure S1.

in mammalian cell division were provided in our previous report that inhibition of glucosylceramide synthase (GCS) causes cytokinesis failure. Chemical inhibition of GCS versus RNAi knockdown resulted in changes in different lipid species, highlighting the high plasticity of lipid regulation in response to different perturbation methods (Atilla-Gokcumen et al., 2011; Eggert et al., 2006a; Castoreno and Eggert, 2011). The specific ceramides changed upon GCS inhibition, however, mostly differ from the species upregulated in dividing cells, which are ceramides with long fatty acid side chains. Some of the largest relative lipid accumulations in dividing cells were dihydroceramides (diH-ceramides, Table 1 and Figure S1). These lipids are expressed at very low levels in nondividing cells and have been studied little because it was assumed that they were inert precursors during ceramide synthesis. Our findings suggest a broader role. Our study links several specific and unusual lipids to cell division and, more broadly, reports a cell-cycle-dependent regulation of the global lipidome during a basic biological process.

Dividing Cells Have Distinct Mechanical Properties

Having uncovered the high precision that cells use to regulate their lipidomes, we wanted to better understand whether the lipids that we identified had any particular physical properties that might give us hints about their biological functions. In cells, lipids function in complex environments that include different proteins and, if in the plasma membrane, extracellular matrix and cell surface carbohydrates, making it challenging to dissect the contributions of membranes and the lipids themselves. We used atomic force microscopy (AFM) in its force spectroscopy mode to first investigate the mechanical properties of live cells at 37°C. At relatively low forces and low indentation depths, force-spectroscopy AFM has been used successfully to measure the elasticity of a range of different cell types and experimental environments (Harris and Charras, 2011; Kasas et al., 2013; Stewart et al., 2012). For example, elasticity measurements have been applied to study cell division and spreading (Matzke et al., 2001; Pietuch and Janshoff, 2013), and we use this approach here to investigate the properties of cells in which lipid biosynthesis has been perturbed (see below and Figure 3F). To gain more insight into the full range of mechanical stabilities of dividing cells, we turned to a less common approach: we expanded the traditionally sampled range of forces to higher values, spanning 10–250 nN and compressed the cell until the plasma membrane was penetrated just before the tip reached the hard substrate (Hategan et al., 2003). Applying such high forces deforms the entire cell, pushing the AFM probe through a variety of cellular structures and compartments until the membrane is broken. The rupture of the plasma membrane is recognized by a breakthrough jump in the force-distance curve, representing a clear molecular fingerprint (Garcia-Manyes et al., 2005a, 2010), which is similar in cells and in supported lipid bilayers (Figure 2). We based this work on a precedent (Yokokawa et al., 2008), wherein it was demonstrated that the "rip" encountered at high forces during indentation corresponds to the real penetration of the probe through the plasma membrane (see Extended Experimental Procedures for more details). We found that much higher forces (3-fold increase) were required to reach this breaking point in

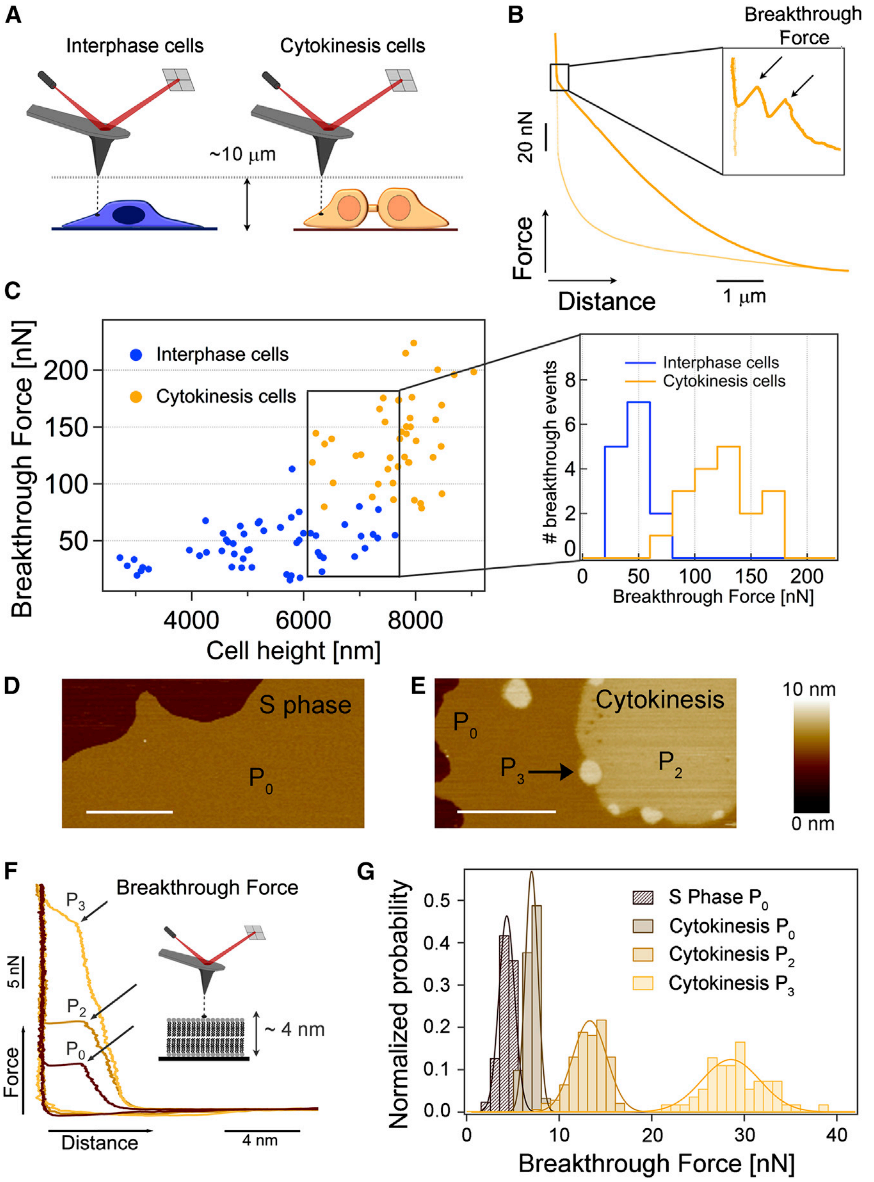

Figure 2. The Mechanical Resistance of Cytokinesis-Associated Membranes Is Higher Both in Live Cells and in Supported Lipid Bilayers Measured In Vitro

(A) Schematics of a force spectroscopy experiment on an interphase (nondividing) and cytokinesis (dividing) live cell.

(B) Typical force-distance curve conducted on a live HeLa cell during cytokinesis. In a typical cycle, the cantilever tip approaches the cell membrane and applies an increasingly higher pushing force that deforms the cytoplasm. Once the cantilever reaches a position close to the stiff substrate, it is able to penetrate through the two lipid membranes. This breakthrough event is marked by two consecutive discontinuities in the force-extension trace (arrows in inset). The cantilever is then retracted to the initial position (light-yellow line).

(C) Scatterplot of the first membrane breakthrough force versus the cell height for interphase (blue, 26 independent cells) and cytokinesis cells (yellow, 17 independent cells) in cytoplasmic areas. Similar experiments performed in the nuclear areas are shown in Figure S2. The plot shows that, although the breakthrough force increases with the cell height, dividing cells exhibit markedly higher breakthrough forces. Selecting a comparable range of cell heights (6–7.6 μm), the mechanical resistance of cytokinesis cells is significantly higher (inset in C, 125 ± 30 nN and 48 ± 14 nN breakthrough force for dividing and interphase cells, respectively) with >99.99% confidence (Student's t test). Though the error in comparing values between dividing and nondividing cells is small, there is an additional potential error in the absolute values reported due to limitations in the size of the cantilevers available for these experiments (see Extended Experimental Procedures for a discussion).

(D and E) Lipids derived from cells at S phase and cytokinesis form supported lipid individual bilayers that exhibit distinct morphologies as revealed by tapping mode AFM topographical images.

(D) Supported lipid bilayers composed of S phase lipids exhibit a continuous P_0 phase.

(E) By contrast, in the case of lipids extracted from cytokinesis cells, three main phases of increasing height are observed (respectively, P_0, P_2, and P_3). Scale bar, 1 μm.

(F) Force-distance curves on both supported lipid bilayers revealed distinct mechanical stabilities.

(G) The mechanical resistance of lipid bilayers formed from S phase cells (P_0) yielded a mean breakthrough force of 3.87 ± 0.84 nN (n = 900), obtained by Gaussian fits to the data. In the case of the cytokinesis sample, both the P_2 phase (12.93 ± 1.83 nN, n = 137) and the P_3 phase (28.3 ± 3.17 nN, n = 54) revealed a much higher mechanical stability than the matrix bilayer phase, P_0 (6.5 ± 0.68 nN, n = 90).

Note that the nomenclature defining each of the phases is arbitrary yet consistent throughout the paper. For detailed descriptions of all phases and the height profile of (E), see Figure S2.

dividing versus nondividing cells of comparable heights (Figures 2A–2C), suggesting that both the cell body and the plasma membrane have different mechanical properties in dividing cells. The same trend was observed in the nuclear regions (Figure S2A and S2B), albeit shifted to higher force values. Much of the increased stiffness in dividing cells is undoubtedly due to the cytoskeleton, which has well-documented and essential roles in the division process. However, our experiments suggest that the properties of the plasma membrane also need to be different in dividing cells to be able to withstand substantially higher forces applied during force spectroscopy.

Lipids Isolated from Dividing Cells Have Distinct Morphological and Mechanical Properties

The mechanical resistance that we found in the plasma membrane of dividing cells is likely due to the combined action of membrane proteins, cell surface glycans, and lipids. The full complement of membrane proteins is not known, but we show

Table 2. Cytokinesis Hits from an RNAi Screen of Lipid Biosynthetic Enzymes

Gene Symbol	Predicted Lipid Product	% Multinucleated
SMPD4	*ceramide*	*51.7 ± 4.9*
CERS4	*diH-ceramide/ceramide*	*24.2 ± 2.8*
GALC	*ceramide*	*22.5 ± 6.3*
DGAT2	*triacylglycerol*	*21.9 ± 6.6*
SERINC4	*phosphatidylserine/* 3-ketodihydrosphingosine	*17.7 ± 2.8 (96h)*
LSS	lanosterol	17.6 ± 1.9
SMPDL3A	*ceramide*	*16.1 ± 3.2 (96h)*
CH25H	*25-hydroxycholesterol*	*14.9 ± 3.4 (96h)*
SERINC1	*phosphatidylserine/* 3-ketodihydrosphingosine	*14.4 ± 0.3 (96h)*
ACSL5	acyl-CoA	13.9 ± 2.3
ALOX12B	12(R)-HPETE	13.8 ± 5.6
PIKFYVE	PI(5)P/PI(3,5)P2	13.4 ± 1.4
GLB1	*glucosylceramide*/GM2	*13.3 ± 4.0*
LYPLA2	glycerophosphocholine/ glycerophosphoethanolamine	13.2 ± 2.8
GAL3ST1	sulfatide/ digalactosylceramidesulfate	13.0 ± 0.8
SCD	oleoyl-CoA/ oleoyl-[acyl-carrier protein]	12.2 ± 0.7
ABHD5	*phosphatidic acid*	*11.6 ± 2.0*
MTM1	*PI* /PIP/I(1)P	*11.5 ± 3.1 (96h)*
ST6GALNAC6	GD1α/GQ1bα/GT1aα	11.4 ± 2.7 (96h)
ST8SIA5	GD1c/GT1a/GQ1b/GT3	11.1 ± 3.8
LCAT	1-acylglycerophosphocholine/ cholesteryl ester	10.8 ± 1.9
PTPLB	VLC trans-2,3-dehydroacyl-[acyl-carrier protein]/trans-2, 3-dehydroacyl-CoA	10.2 ± 0.9
CERS2	*diH-ceramide/ceramide*	*10.0 ± 1.0*
Nontargeting siRNA control		2.3 ± 0.8

Enzymes shown in *italics* are predicted to process lipid families that accumulate in cytokinesis and/or midbody samples (Table 1). Predicted lipid products were identified using the KEGG database (http://www.genome.jp/kegg/). See Table S2 for a full list of the RNAi library and the RNAi screen section in the Extended Experimental Procedures for additional details. The average percentage of multinucleated cells, as well as the standard deviation between the four experiments, is shown (two independent experiments with two time points each: 72 and 96 hr). A minimum of 100 cells per case were counted.

here precisely which lipids change in dividing cells. If the lipids themselves have physical properties that might influence their function, these could include a predisposition to organize into different domains, possibly recruiting other lipids and/or proteins to these domains in cells. In an attempt to identify a potential contribution of lipids to the overall mechanical stability of S phase and dividing cells, we again used AFM to test the topographic and mechanical properties of supported lipid bilayers from isolated lipids at very high spatial resolution (El Kirat et al., 2010; Sackmann, 1996) (Figures 2D–2G). Because the different membrane compartments in cells are very dynamic and we have an incomplete understanding of the membrane proteins involved that could potentially be used as markers for a membrane fraction's origin especially in dividing versus nondividing cells, it is not possible to isolate only plasma membrane in sufficient purity to allow meaningful comparisons. We therefore analyzed total lipids isolated from cells under the same conditions as the samples used for LC-MS, allowing a direct comparison between the identities of the lipid mixtures and their properties.

Although the differences in lipids that we observed by LC-MS were large for specific lipids, the total change was quite small relative to the cellular lipidome (for example, the total amount of ceramides doubled, but only ~2% of lipids in HeLa are ceramides). Surprisingly, such a small detected change in the lipid composition had a significant effect on the topographic properties of supported lipid bilayers formed from S-phase- and cytokinesis-derived membranes: supported lipid bilayers formed from S phase cells were mostly uniform (presenting only the matrix phase "P_0"; Figures 2D and S2C), whereas lipids from dividing cells were more likely to separate into three distinct phases (P_0, P_2, and P_3) (Figure 2E). To test whether such a distinct lateral molecular arrangement of the lipids also had an effect on the bilayers' mechanical properties, we conducted force spectroscopy experiments on both samples (Figures 2F and 2G) (Garcia-Manyes and Sanz, 2010). Whereas bilayers corresponding to S phase cells (P_0) exhibited moderate mechanical stability of ~4 nN (Figure 2G), the distinct phases found in the bilayers formed from cytokinesis cells exhibit increasingly larger forces required to puncture the membrane, culminating in the P_3 phase (Figures 2E and 2G; see Figure S2 for further description of the different phases). The P_3 phase, which was observed in about 20% of samples (Figure S2D), was dramatically stiffer, with an associated mechanical resistance of ~28 nN. These observations on supported lipid bilayers suggest that some of the mechanical properties that we observed in cells could be related to the physical properties of the lipids themselves, functioning in conjunction with membrane proteins.

RNAi Knockdown of Lipid Biosynthetic Enzymes Causes Cell Division Defects

To get a better understanding of the lipids' potential biological roles, we perturbed lipid levels in cells and evaluated the resulting phenotypes. In the absence of techniques for lipids comparable to genetic knockouts or knockdowns, inhibition of lipid biosynthesis is one of the few ways in which lipid levels in cells can be manipulated. We used RNAi to systematically perturb the biosynthesis of different lipid families. We designed a custom library targeting 244 lipid biosynthetic enzymes and screened this library for cytokinesis inhibition in HeLa cells. Knockdown of 23 genes caused cytokinesis failure (Tables 2 and S2). Although there was a range across different lipid classes, 11 out of 23 are involved in sphingolipid metabolism, which is in excellent agreement with our LC-MS data. Some of these enzymes are predicted to process highly glycosylated sphingolipids (GLB1, ST6GALNAC6, and ST8SIA5), which were not detected in our lipidomic analysis using

standard LC-MS parameters. These data partially correlate with the literature. It was shown in sea urchin eggs that cholesterol and the glycosphingolipid GM1 accumulate in an equatorial band coupled to contractile ring formation and assembly (Ng et al., 2005). GM1 is one of GLB1's substrates, and both ST6GALNAC6 and ST8SIA5 process GD1a, a direct derivative of GM1.

The broad importance of different lipids and membrane compartments in the cell division process is highlighted by our identification of enzymes associated with a variety of different pathways and predicted functions and localizations. For example, two enzymes are predicted to process sterol derivatives (LSS, CH25H). Other enzymes are implicated in the glycerophospholipid and glycerolipid pathways (LYPLA2, LCAT, SERINC1 and 4, DGAT2, and ABHD5), and two enzymes (PIKFYVE and MTM1) process phosphatidylinositols, which are known to be involved in cytokinesis (Echard, 2012). In addition, we identified enzymes predicted to be involved in the biosynthesis, metabolism, or elongation of fatty acids: ACSL5, ALOX12B, SCD, and PTPLB. Some of these enzymes may be involved in the synthesis of precursors of the lipids that we identified by LC-MS.

Several of the most penetrant RNAi phenotypes are caused by single enzymes that are part of multimember families, for example, ceramide synthases (six enzymes) or neutral sphingomyelinases (four enzymes in mammals). The field is only just beginning to appreciate the complexity of lipid biosynthesis and the level of crosstalk between different biosynthetic routes and enzymes. Many biosynthetic enzymes have predicted substrate and product families, but the nature of specific lipids involved and the factors regulating the enzymes and therefore lipid production are only known in very few cases (Hannun and Obeid, 2011). For example, a biochemical analysis of different ceramide synthases showed that different enzymes preferentially process different side-chain-length ceramides and that their expression is tightly connected (Mullen et al., 2011). We therefore used LC-MS to analyze the lipid composition of three top RNAi hits: SMPD4, GALC, and DGAT2 (Figure S3A and Table S3). DGAT2 is an acyltransferase that catalyzes the terminal step in triacylglycerol synthesis, GALC catalyzes the conversion of galactosylceramide into ceramide, and SMPD4 is predicted to convert sphingomyelin into ceramide (Figure S3B). Unexpectedly, knockdown of SMPD4 did not result in accumulation of substrate or depletion of product, as would be predicted by classical enzymology, but instead caused accumulation of specific side-chain ceramides (product) and glycosylated ceramides. This is likely due to cells adjusting their substrate specificities and activating alternative lipid biosynthesis feedback loops over the 3 day duration of the RNAi experiment. GALC RNAi also caused accumulation of specific side-chain hexosylceramides. DGAT2's lipid profile is discussed further in the section on midbodies because we found that it is involved in the metabolism of a lipid accumulated at the midbody.

RNAi Knockdown of Key Lipid Biosynthetic Enzymes Causes Mechanical Defects in Cells

The overall cellular consequences of SMPD4, GALC, and DGAT2 knockdown were similar and connected to defects in the cytoskeleton, the main driver of cell mechanics. To varying degrees, all three RNAi treatments caused a significant delay during metaphase in mitosis (Figures 3A and 3B), followed by cell division failure at different stages, mostly coupled with unusual membrane blebbing (Figures 3A and S3D). The morphologies of interphase cells as well as the actin cytoskeleton were also changed, including an increase in the footprint of cells (Figures 3D and 3E). Because these phenotypes suggest defective processing of mechanical signals, we tested whether mechanical integrity was affected in cells in which our top hit SMPD4 was knocked down. Using AFM at forces lower than 0.6 nN (as opposed to the high forces needed to break through membranes discussed above) to measure elasticity, we found that SMPD4 RNAi cells are 4-fold stiffer than control cells (Figure 3F). These data show a clear mechanical role for lipids, either directly or by causing changes to the cytoskeleton. Because SMPD4 RNAi is accompanied by changes in the actin cytoskeleton (Figure 3D), in this case it is likely that the effects exerted by lipid changes cause cytoskeletal defects rather than structural roles of the lipids themselves.

Dividing Cells Regulate the Lipid Composition of the Midbody

Membranes are particularly important in the midbody, where final cleavage takes place, because they are needed to seal the plasma membrane after fission. Super-resolution imaging shows that ceramide-positive vesicles and the glycosylated sphingolipid GM1 localize to different regions of the midbody (Figure 4A). Ceramide vesicles do not substantially overlap with endosomal proteins known be involved in cytokinesis (FIP3-RAB11 and ESCRTIII; Figures 4B and 4C), suggesting that they participate in an as yet unknown mechanism. Membrane trafficking is reduced during mitosis and is thought to increase again during cytokinesis. Thus, a specific lipid composition at the midbody might be regulated both by specific trafficking and by local synthesis. We therefore tested whether cells regulate their lipid composition at the midbody. Midbody-rich fractions can be purified biochemically, and their proteome has been determined. To isolate midbodies with intact membranes, we adapted previous protocols (Mullins and McIntosh, 1982; Skop et al., 2004) and compared lipids extracted from cells in cytokinesis and midbodies (Figure 5). To control for the copurification of nonmidbody lipid structures, we also compared the lipidome of midbodies to lysate from nonsynchronized cells that was subjected to the same purification protocol (Figure 5D). As with dividing cells, we found that a small number of lipids with very specific structures strongly accumulated in midbodies (Tables 1 and S1 and Figure 1). Five out of nine lipids that accumulate at midbodies also accumulate during division, and all five lipids are sphingolipid derivatives with specific fatty acid chains, suggesting that long-chain dihydroceramides' and ceramides' effects are specific to the abscission site.

Several lipid species, including a rare TAG (16:1, 12:0, 18:1) (Figure S4), accumulate in midbodies but were not upregulated in dividing cells, suggesting that they are always present in cells and specifically localize to the midbody. TAGs are the primary unit of energy storage in eukaryotic cells. Whereas the lengths of fatty acid chains in TAGs vary significantly based on the cells'

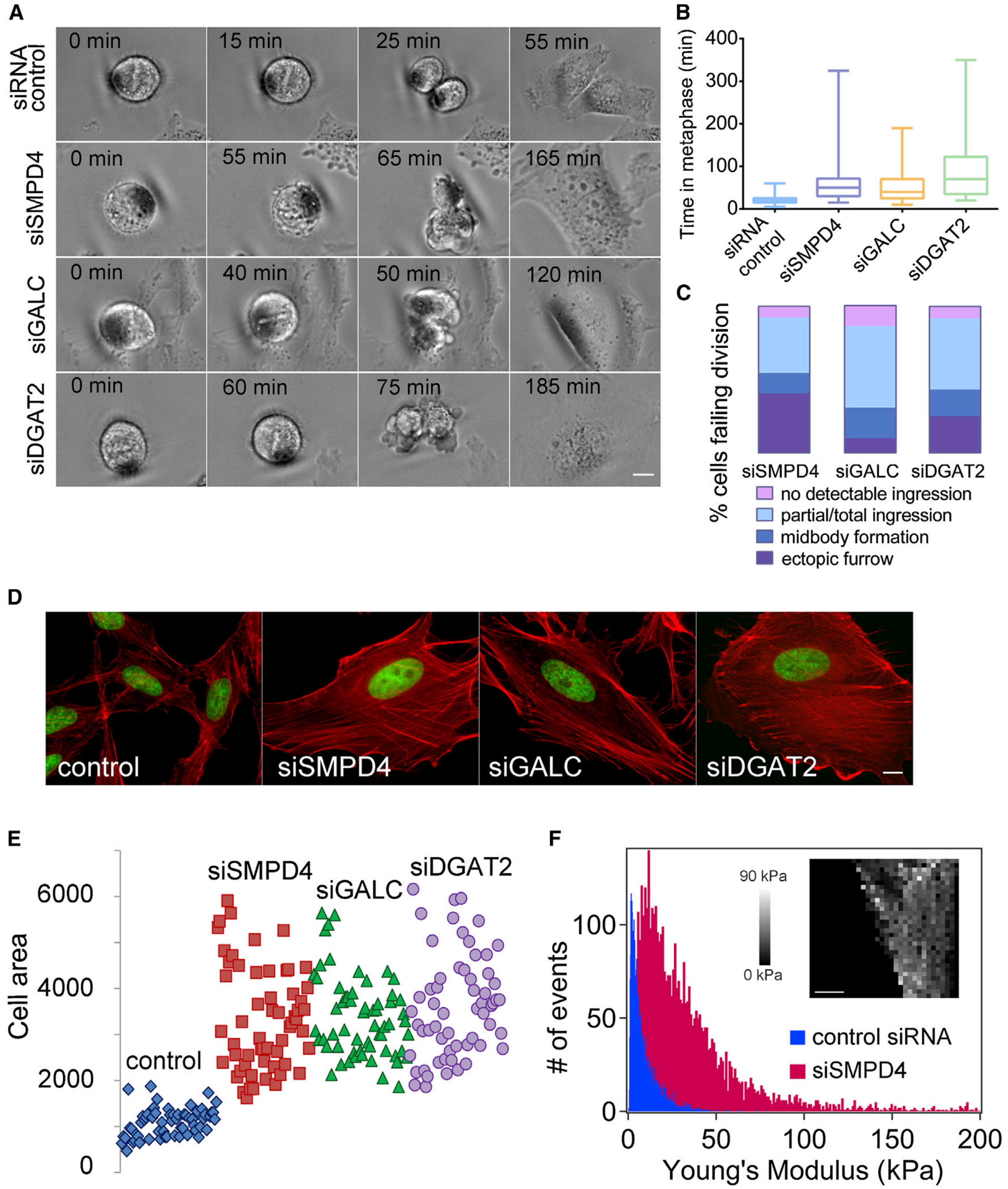

Figure 3. RNAi Knockdown of Lipid Biosynthetic Enzymes Causes Cell Division Defects and Cytoskeletal Changes in Interphase Cells

(A) Still images from time-lapse movies of dividing cells for control, SMPD4, GALC, and DGAT2 siRNA treatments. Movies are available in the Supplemental Information. Scale bar, 10 μm.

(B) Box plots of metaphase delays caused by siSMPD4, siGALC, and siDGAT2 treatments, respectively (average ± SEM), 58 ± 4.6, 56.5 ± 5.4, and 90.6 ± 9 min. The time in metaphase for all RNAi-treated cells was significantly higher than the control (20.4 ± 1 min) with a > 99.99% confidence (t test). A minimum of 70 cells from 3 independents experiments for each case were analyzed.

(C) SMPD4, GALC, and DGAT2 siRNA treatments result in different cytokinesis failure phenotypes. Examples for each phenotype are shown in Figure S3D. Ectopic furrow refers to both successful and unsuccessful abscissions. A minimum of 70 cells from three independent experiments for each case were analyzed.

(D–F) SMPD4, GALC, and DGAT2 siRNA treatments result in altered cell shape and actin morphology.

(D) Representative images are shown for control and siRNA-treated cells. F-actin is shown in red (phalloidin staining) and DNA in green (DAPI staining). Scale bar, 10 μm.

(E) Quantification of the cell area for control cells (1088 ± 39 μm^2, average ± SEM) and different siRNA-treated cells (respectively, for siSMPD4, siGALC, and siDGAT2: 3331 ± 143, 3386 ± 113, and 3617 ± 144 μm^2). The area was measured in at least 60 mononucleated cells per treatment and was significantly higher in

(legend continued on next page)

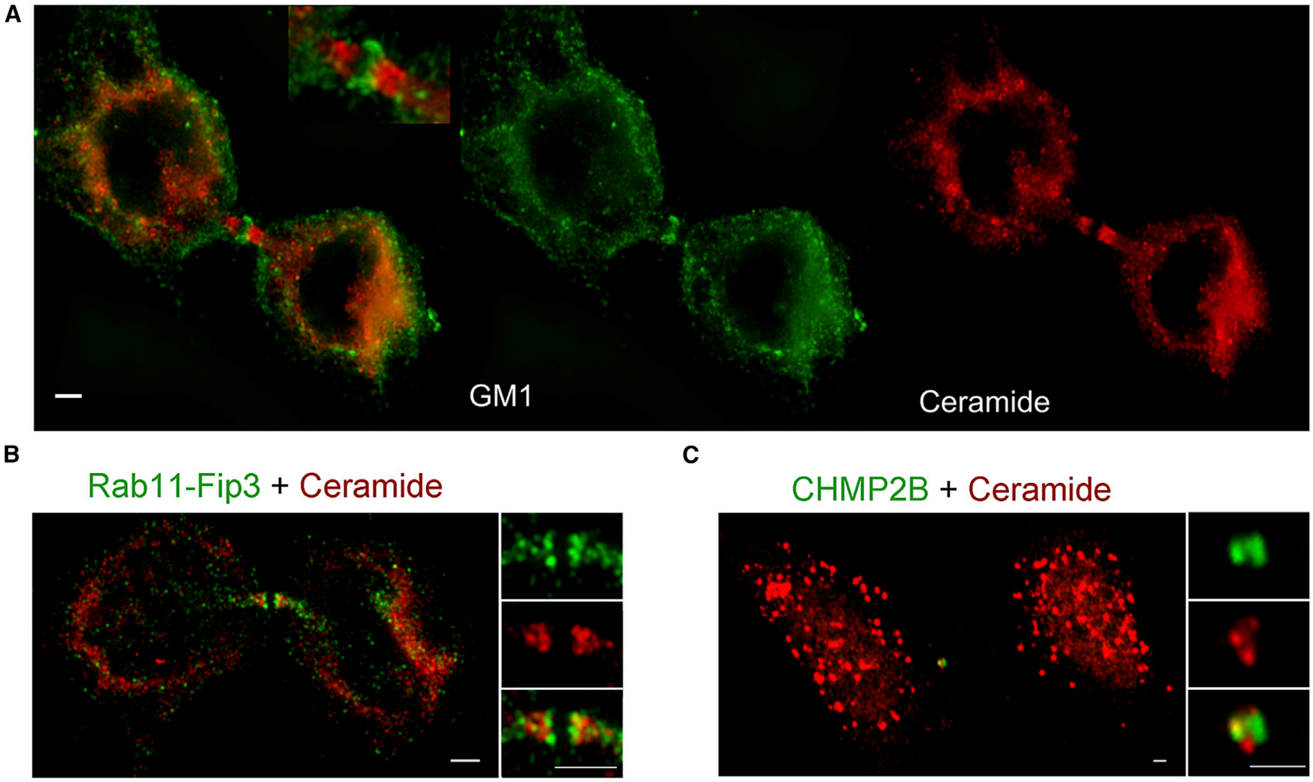

Figure 4. Ceramide-Containing Vesicles Localize to the Midbody

(A) Different lipids show differential localization to division sites. The glycosphingolipid GM1 (green, visualized by cholera toxin) and ceramide (red, pan-ceramide antibody staining) at the midbody are shown. Images are Z projections and were acquired with a N-SIM super-resolution microscope. Inset shows 2× zoom of the midbody area. Scale bar, 2 μm.

(B and C) Ceramide antibody staining only partially overlaps with endosomal proteins known to be involved in cytokinesis: FIP3-RAB11 (B) and CHMP2B (C). The images are Z projections acquired with an N-SIM super-resolution microscope (B) and a confocal microscope (C). Scale bars, 2 μm.

repertoire, longer-chain fatty acids (14–20 carbons) are usually preferred in eukaryotic cells. Our TAG species is unusual because it contains a 12 carbon chain. Although cells make thousands of different TAGs, the biological functions of individual species are not well studied and neither are the selectivity or specificity of several different enzymes that synthesize TAGs (Coleman and Mashek, 2011). DGAT2, a TAG-synthesizing enzyme (Figure S3C), was a top hit in our RNAi screen (Figures 3A–3C). MS analysis showed that several lipids were changed in DGAT2 RNAi cells, including TAG (16:1, 12:0, 18:1) (Figure S3A), suggesting that DGAT2 is involved in the metabolism of this lipid.

A specific phosphatidylserine also accumulated at midbodies. Phosphatidylserines mostly reside in the plasma membrane and are present in the inner leaflet in the resting state of the cell. Synthesized in the endoplasmic reticulum, phosphatidylserines are transported through different organelles and to the plasma membrane and can also be flipped from the inner leaflet to the outer leaflet of the plasma membrane when necessary. This dynamic nature is important for biological processes, including apoptosis and autophagy (Kay and Grinstein, 2011). Biological functions for specific unsaturated long-chain phosphatidylserine species, such as the one that we identified, have not been reported.

In addition to the more unusual lipid species that we observed in dividing cells and at midbodies, we also found two common lipids: (16:0/16:0) phosphatidic acid accumulated in midbodies and (16:0/18:0) phosphatidylinositol in dividing cells. Both fatty acid configurations represent major subspecies within these lipid families. As a major constituent of the plasma

all RNAi-treated cells compared to control, with a >99.99% confidence (t test). Targeted lipid analyses of the RNAi samples are reported in Table S3 and Figure S3A.

(F) Histogram of cell stiffness (Young's modulus values, see Extended Experimental Procedures) measured on control and SMPD4 siRNA-treated cells. A total number of 4,098 indentations (21 cells) and 4,314 indentations (18 cells) were performed on control siRNAi and siSMPD4-treated cells, respectively. The average value for siSMPD4 cells is significantly higher (35 ± 8.8 kPa, average ± SEM) than siRNA control cells (8.3 ± 1.9 kPa), with a >99.95% confidence. (Inset) Typical 32 × 32 force-volume map measured on a siSMPD4 cell. The topography of the force-volume corresponds to an arbitrary color scale of the Young's Modulus. Scale bar, 10 μm.

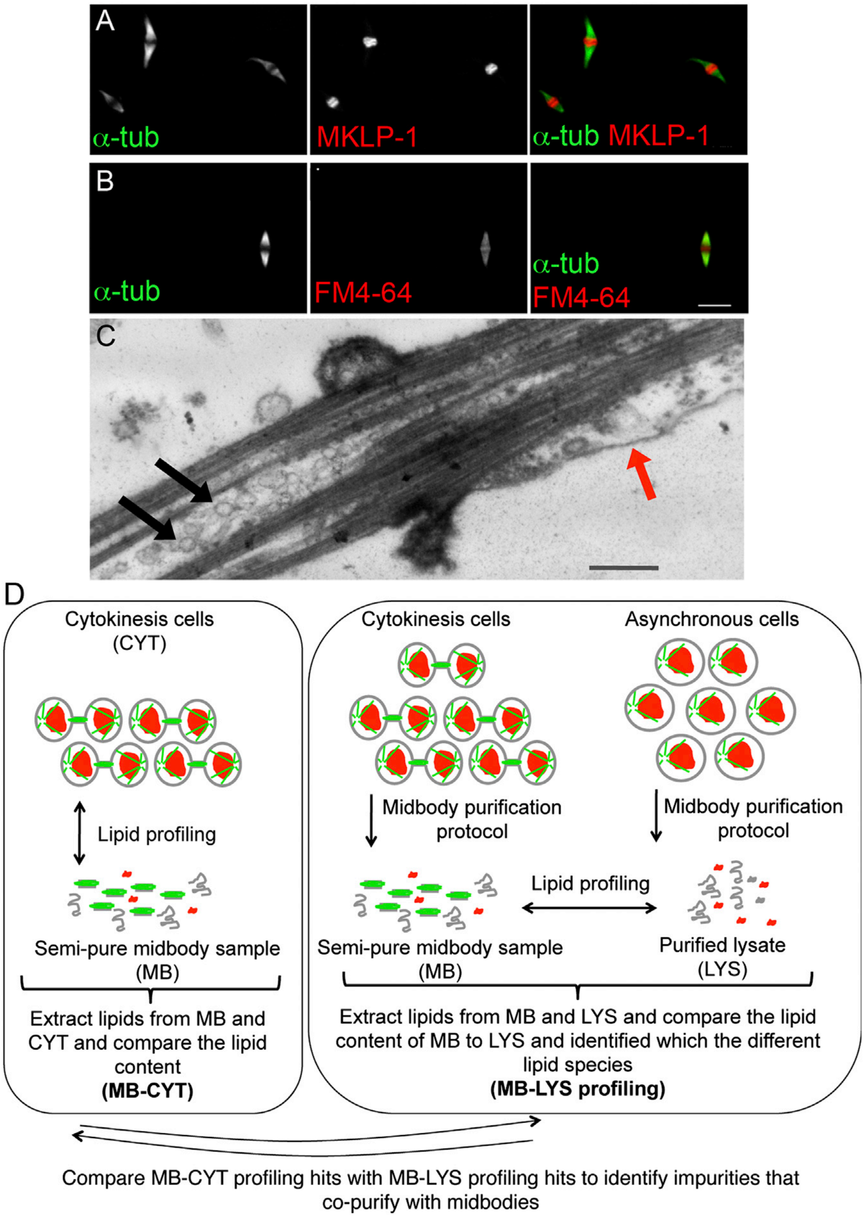

Figure 5. Midbody and Membrane Markers Are Correctly Localized in Isolated Intact Midbodies

(A and B) Isolated midbodies were stained with α-tubulin, MKLP-1, a marker for midbodies (A), and FM4-64, a membrane marker (B). Scale bar, 5 μm.

(C) Representative electron micrograph shows an isolated midbody with intact membrane structures. Black arrows show vesicles inside, and red arrow shows plasma membrane that surrounds the midbody. Scale bar, 500 nm.

(D) Schematic of the experiments that we conducted to exclude from our LC-MS analysis membrane impurities that copurify with midbodies. We obtained and compared the profiles of midbodies versus cytokinesis (MB-CYT) and midbody versus purified lysate (MB-LYS). The hits from MB-CYT and MB-LYS profiling were compared. MB-CYT hits were checked in MB-LYS profiling. Lipids that were unchanged in MB-LYS profiling (i.e., found in lysate from asynchronous cells in equal amounts) were eliminated, as they are most likely impurities that copurified in both samples. Membrane structures, microtubules, and DNA are cartooned in gray, green, and red, respectively.

membrane, phosphatidic acids are important signaling molecules and precursors for a diverse group of lipids, including phosphatidylserines and phosphatidylinositols. Phosphatidylinositols are a constituent of the inner leaflet of the plasma membrane. They can be glycosylated and form glycosylphosphatidylinositol (GPI) anchors, which are important post-translational protein modifications. They are also precursors for phosphatidylinositol phosphates, important signaling molecules that actively participate in cell division (Echard, 2012). Much of lipid signaling is regulated through localization of lipids to specific membrane structures, often vesicles. Our data show that cells not only specifically regulate when they synthesize specific lipids, but also where they are localized within larger structures of the cell.

There Is High Correlation between Lipid Species Identified by LC-MS and RNAi Screening

It has become clear in recent years that the lipid composition of different cell types can vary substantially for reasons that are only beginning to be understood (Gerl et al., 2012). The work that we report here was done in HeLa, and we expect that the extent and/or identity of lipid changes in other dividing cells may vary depending on the physiology of the cell type. We show, however, excellent agreement between the lipid families targeted by the biosynthetic enzymes that scored in our RNAi screen and the lipids identified in the unperturbed LC-MS analysis (overlap is shown in italics in Table 2). Twelve out of 23 enzymes identified in our RNAi screen are predicted to process lipids found accumulated in cytokinesis cells and/or at the midbody, eight of which belong to the nine strongest RNAi hits. Reaching the same results by two complementary approaches strongly suggests that the lipids we identified by both approaches have biological significance.

DISCUSSION

We have shown here that HeLa cells actively regulate the production and localization of lipids during cell division and that cells display high specificity in the chemical structures of these lipids. Our analysis methods were comparative

(cytokinesis versus S phase cells and midbody versus purified lysate), allowing us to observe dynamic changes between the different states we analyzed. Several of the lipids that had previously been reported to play a role during cytokinesis, including the phosphatidylinositol phosphates (Emoto et al., 2005; Field et al., 2005), did not score in our LC-MS analysis. This is, in part, due to our inability to detect large lipids like PIP3 with the extraction conditions and the MS ionization method that we used (ESI). We could, however, detect PIP2, which was unchanged, likely due to its constant presence during the cell cycle, as would be expected for a signaling lipid that regulates multiple processes. Along this continuum and as with many proteins involved in cell division, some of the lipids reported here appear to also be involved in other cytoskeletal processes; for example, GALC, SMPD4, and DGAT2 RNAi interphase cells have altered actin morphologies (Figures 3D and 3E). The required presence of some lipids throughout the cell cycle explains why we observed very good, but not complete, correlation between the dynamic lipid changes identified by LC-MS and the biosynthetic enzymes scored in our RNAi screen. It is likely that several of the biosynthetic enzymes that did not have matching lipid changes are involved in the metabolism of lipids that are constantly present.

Our analyses showed not only that cells precisely regulate the timing of when they synthesize specific lipids, but also that they specifically regulate their localizations to the midbody. It is beginning to be appreciated that different membrane compartments such as some endosome species, for example, have distinct lipid compositions (Bissig and Gruenberg, 2013). We focused our analysis on the lipid composition of midbodies, both because they are crucial sites of cell fission and because we could purify them, and appropriate controls, in sufficient quantity and acceptable purity. We were interested to note that there was only partial overlap between the lipids accumulated in dividing cells and at midbodies, suggesting that several of the lipids accumulated in dividing cells have functions that are independent of the final division site, possibly during earlier steps of division. Conversely, several midbody lipids did not accumulate in dividing cells, suggesting that they have additional roles during the cell cycle. A finer dissection of the regulation of lipid localization, for example to the plasma membrane or to different vesicle types, would provide further functional information, but is currently limited by the ability to biochemically fractionate different compartments with sufficient purity. As our understanding of the protein requirements of different membrane compartments grows, it should become possible to use the protein markers as guides for more detailed lipidomic studies.

Despite decades of intense study predominantly focused on the proteins of the actin cortex located just beneath the plasma membrane (Eggert et al., 2006b), we still do not have a comprehensive understanding of the mechanics underlying the cell division process. For example, local tension changes of the actin cortex both at the cleavage furrow and at the opposing poles are important for successful division (Guha et al., 2005; Murthy and Wadsworth, 2005; Sedzinski et al., 2011). A recent report showed that a release of membrane tension triggered the assembly of the ESCRT fission complex during abscission (Lafaurie-Janvore et al., 2013). These intriguing observations suggest that there may well also be mechanical roles for membranes. These roles could be structural or could involve signaling to the cytoskeleton. We present here evidence suggesting that both occur, and we begin to tease apart the contributions made by lipids and membrane proteins. Lipids are involved in signaling by affecting specific membrane proteins or by assembling multiprotein and multilipid signaling platforms. We show that SMPD4 RNAi makes cells stiffer and causes defects in the actin cytoskeleton, likely because changes in the lipid composition cause the transmission of signals to the cytoskeleton.

We also present evidence for possible structural roles of lipids during division. Dividing cells are substantially more resistant to high forces applied by force spectroscopy than are nondividing cells, suggesting that their membranes have changed to adapt to mechanical stress during division. Although some of these properties are no doubt due to membrane proteins or lipid/protein signaling platforms, we now know precisely which lipids change in dividing cells, allowing us to consider whether the lipids themselves also might contribute. As it is not possible to use measurements in live cells to dissect the contributions of lipids versus membrane proteins, the cytoskeleton, or the cytoplasm (Moeendarbary et al., 2013), we used force spectroscopy on supported lipid bilayers isolated from dividing cells. We found an increased tendency toward more rigid domain formation, including the extraordinarily rigid P_3 phase. High-resolution AFM studies on lipid phase behavior are usually performed with carefully controlled synthetic membranes (Sullan et al., 2009a, 2009b). Here, though we know which lipids differ between the two samples, both are complex mixtures of total lipids extracted from cells. This allows interactions between lipids normally segregated in different cellular membrane compartments. Despite this dilution of possible naturally occurring interactions in both samples, we observe an increased tendency to form distinct phases in lipids extracted from dividing cells (Figures S2A and S2B), and we were intrigued by the surprisingly distinct change in the physicochemical properties of the supported lipid membranes. Due to the artificial conditions of the AFM experiment on supported lipid bilayers, we would not expect to observe identical domains in cells. However, in the absence of other techniques to study the role of lipids, it is tempting to speculate that the ability of cytokinesis lipids to form rigid phases in vitro could contribute to the extraordinary mechanical resistance that we observed in live dividing cells under high forces. In parallel with the development of methods to study the functions of lipids directly in the cell membrane, advances in biophysical techniques to study cell mechanics with enhanced resolution will be needed to conclusively understand the mechanical roles of different cellular and membrane compartments during division.

The order of magnitude of lipid diversity (10,000 s) approaches that of proteins, but the field is only beginning to appreciate this diversity. We show that dividing cells display exquisite specificity in their lipid composition, and many of the lipids that we identify are quite rare species that have not been assigned biological functions. Our discovery of the high precision that cells use to regulate their lipidome during the cell cycle suggests that these lipids play key roles in the division process. Although understanding the detailed roles of the lipids that we identified will

require further technological advances in lipid biology, we have made a start by showing that lipids contribute to the structural integrity of dividing cells and are involved in the transmission of signals. We have also identified strategies to perturb lipid levels by using RNAi to identify which biosynthetic enzymes are required for division, and we have described the cellular consequences of lipid perturbations. This work makes it clear that lipids play active and essential roles in a fundamental biological process and significantly strengthens the newly emerging paradigm that specific lipids within lipid families have specific functions.

EXPERIMENTAL PROCEDURES

Detailed information on the materials used can be found in the Extended Experimental Procedures section, as well as detailed procedures for cell culture methods and the RNAi screen.

S Phase Arrest

HeLa S3 cells were arrested at S phase by using thymidine. Cells were plated into complete DMEM containing 4 mM thymidine at a final concentration of 4×10^5 cells/ml in 10 cm dishes for 24 hr (in 10 ml). Cells were then washed twice with 5 ml PBS and were harvested using a cell scraper in 5 ml PBS. Cells were pelleted by centrifugation at 200 × *g*, and the pellets were stored at −80°C for S phase cells.

Cytokinesis Arrest

For a full description of the cytokinesis arrest, see the Extended Experimental Procedures. In brief, 100 nM nocodazole treatment for 12 hr yielded a highly mitotic population of HeLa S3 cells. Mitotic cells were collected by mitotic shake-off, and cells were replated after several washing steps. Cells were incubated for 2 hr, allowing them to synchronously release to cytokinesis, and Taxol was added prior to harvesting if cells were carried forward for midbody isolation.

Midbody Isolation

For a full description of the cytokinesis arrest, see the Extended Experimental Procedures. In brief, the first step of the isolation is the mechanical lysis of cytokinesis cells in Mb buffer using a 27 G1/2 needle. The resulting solution was vortexed in the presence of glass beads followed by the addition of micrococcal nuclease. Next, the solution was clarified by consecutive short spins to remove unlysed cells and larger structures in the lysate. The concentrated midbody-containing solution was subjected to a brief immunoprecipitation step by using integrin β3 antibody to remove nonmidbody-related membranes. The resulting supernatant was enriched in midbody structures and was used for midbody lipid profiling.

Preparation of Lipid Extracts for LC-MS and Data Analysis

Lipids were prepared according to a previously reported protocol (Saghatelian et al., 2004). A full description of the LC-MS method and lipid species assignments can be found in the Extended Experimental Procedures.

Atomic Force Microscopy

Force spectroscopy measurements using low (~0.6 nN) or high forces (10–250 nN) on cells as well as imaging and force spectroscopy on isolated lipid bilayers were performed using a commercial Dimension Icon AFM instrument (Bruker, Karlsruhe, Germany). Sample preparation, measurement details, and statistical analyses are discussed in the Extended Experimental Procedures.

SUPPLEMENTAL INFORMATION

Supplemental Information includes Extended Experimental Procedures, four figures, three tables, and four movies and can be found with this article online at http://dx.doi.org/10.1016/j.cell.2013.12.015.

ACKNOWLEDGMENTS

We thank Alan Saghatelian, Tim Mitchison, and Xin Zhang for helpful discussions. LC-MS data was acquired on an Agilent 6520 Q-TOF spectrometer supported by the Taplin Funds for Discovery Program at Harvard Medical School (PI, Suzanne Walker). We thank the Nikon Imaging Centres at King's College London and Harvard Medical School for help with light microscopy. We also thank the facilities of the EBN group of the Physics Department of King's College London. This work was funded by NIH grant R01 GM082834, Human Frontier Science Program Young Investigator Award, Marie Curie CIG grant 304137, ERC Starting Grant 306659 (to U.S.E.), the Heinrich Hertz Foundation (to S.S.), a "La Caixa" PhD fellowship (to J.R.-G.), NIH grant R01 GM39565 (M.L.C., awarded to T.J. Mitchison), and Marie Curie CIG grant 293462, BBSRC grant J00992X/1, and EPSRC Fellowship K00641X/1 (to S.G.-M.).

Received: May 16, 2013
Revised: October 16, 2013
Accepted: December 4, 2013
Published: January 23, 2014

REFERENCES

Albertson, R., Riggs, B., and Sullivan, W. (2005). Membrane traffic: a driving force in cytokinesis. Trends Cell Biol. *15*, 92–101.

Atilla-Gokcumen, G.E., Castoreno, A.B., Sasse, S., and Eggert, U.S. (2010). Making the cut: the chemical biology of cytokinesis. ACS Chem. Biol. *5*, 79–90.

Atilla-Gokcumen, G.E., Bedigian, A.V., Sasse, S., and Eggert, U.S. (2011). Inhibition of glycosphingolipid biosynthesis induces cytokinesis failure. J. Am. Chem. Soc. *133*, 10010–10013.

Bissig, C., and Gruenberg, J. (2013). Lipid sorting and multivesicular endosome biogenesis. Cold Spring Harb. Perspect. Biol. *5*, a016816.

Braverman, N.E., and Moser, A.B. (2012). Functions of plasmalogen lipids in health and disease. Biochim. Biophys. Acta *1822*, 1442–1452.

Castoreno, A.B., and Eggert, U.S. (2011). Small molecule probes of cellular pathways and networks. ACS Chem. Biol. *6*, 86–94.

Coleman, R.A., and Mashek, D.G. (2011). Mammalian triacylglycerol metabolism: synthesis, lipolysis, and signaling. Chem. Rev. *111*, 6359–6386.

Echard, A. (2008). Membrane traffic and polarization of lipid domains during cytokinesis. Biochem. Soc. Trans. *36*, 395–399.

Echard, A. (2012). Phosphoinositides and cytokinesis: the "PIP" of the iceberg. Cytoskeleton (Hoboken) *69*, 893–912.

Eggert, U.S., Kiger, A.A., Richter, C., Perlman, Z.E., Perrimon, N., Mitchison, T.J., and Field, C.M. (2004). Parallel chemical genetic and genome-wide RNAi screens identify cytokinesis inhibitors and targets. PLoS Biol. *2*, e379.

Eggert, U.S., Field, C.M., and Mitchison, T.J. (2006a). Small molecules in an RNAi world. Mol. Biosyst. *2*, 93–96.

Eggert, U.S., Mitchison, T.J., and Field, C.M. (2006b). Animal cytokinesis: from parts list to mechanisms. Annu. Rev. Biochem. *75*, 543–566.

El Kirat, K., Morandat, S., and Dufrêne, Y.F. (2010). Nanoscale analysis of supported lipid bilayers using atomic force microscopy. Biochim. Biophys. Acta *1798*, 750–765.

Emoto, K., Inadome, H., Kanaho, Y., Narumiya, S., and Umeda, M. (2005). Local change in phospholipid composition at the cleavage furrow is essential for completion of cytokinesis. J. Biol. Chem. *280*, 37901–37907.

Field, S.J., Madson, N., Kerr, M.L., Galbraith, K.A., Kennedy, C.E., Tahiliani, M., Wilkins, A., and Cantley, L.C. (2005). PtdIns(4,5)P2 functions at the cleavage furrow during cytokinesis. Curr. Biol. *15*, 1407–1412.

Garcia-Manyes, S., and Sanz, F. (2010). Nanomechanics of lipid bilayers by force spectroscopy with AFM: a perspective. Biochim. Biophys. Acta *1798*, 741–749.

Garcia-Manyes, S., Oncins, G., and Sanz, F. (2005a). Effect of ion-binding and chemical phospholipid structure on the nanomechanics of lipid bilayers studied by force spectroscopy. Biophys. J. *89*, 1812–1826.

Garcia-Manyes, S., Redondo-Morata, L., Oncins, G., and Sanz, F. (2010). Nanomechanics of lipid bilayers: heads or tails? J. Am. Chem. Soc. *132*, 12874–12886.

Gerl, M.J., Sampaio, J.L., Urban, S., Kalvodova, L., Verbavatz, J.M., Binnington, B., Lindemann, D., Lingwood, C.A., Shevchenko, A., Schroeder, C., and Simons, K. (2012). Quantitative analysis of the lipidomes of the influenza virus envelope and MDCK cell apical membrane. J. Cell Biol. *196*, 213–221.

Gudejko, H.F., Alford, L.M., and Burgess, D.R. (2012). Polar expansion during cytokinesis. Cytoskeleton (Hoboken) *69*, 1000–1009.

Guha, M., Zhou, M., and Wang, Y.L. (2005). Cortical actin turnover during cytokinesis requires myosin II. Curr. Biol. *15*, 732–736.

Han, X., and Gross, R.W. (2003). Global analyses of cellular lipidomes directly from crude extracts of biological samples by ESI mass spectrometry: a bridge to lipidomics. J. Lipid Res. *44*, 1071–1079.

Hannun, Y.A., and Obeid, L.M. (2011). Many ceramides. J. Biol. Chem. *286*, 27855–27862.

Harris, A.R., and Charras, G.T. (2011). Experimental validation of atomic force microscopy-based cell elasticity measurements. Nanotechnology *22*, 345102.

Hategan, A., Law, R., Kahn, S., and Discher, D.E. (2003). Adhesively-tensed cell membranes: lysis kinetics and atomic force microscopy probing. Biophys. J. *85*, 2746–2759.

Ivanova, P.T., Milne, S.B., and Brown, H.A. (2010). Identification of atypical ether-linked glycerophospholipid species in macrophages by mass spectrometry. J. Lipid Res. *51*, 1581–1590.

Kasas, S., Longo, G., and Dietler, G. (2013). Mechanical properties of biological specimens explored by atomic force microscopy. J. Phys. D *46*.

Kay, J.G., and Grinstein, S. (2011). Sensing phosphatidylserine in cellular membranes. Sensors (Basel) *11*, 1744–1755.

Lafaurie-Janvore, J., Maiuri, P., Wang, I., Pinot, M., Manneville, J.B., Betz, T., Balland, M., and Piel, M. (2013). ESCRT-III assembly and cytokinetic abscission are induced by tension release in the intercellular bridge. Science *339*, 1625–1629.

Matzke, R., Jacobson, K., and Radmacher, M. (2001). Direct, high-resolution measurement of furrow stiffening during division of adherent cells. Nat. Cell Biol. *3*, 607–610.

Meivar-Levy, I., Sabanay, H., Bershadsky, A.D., and Futerman, A.H. (1997). The role of sphingolipids in the maintenance of fibroblast morphology. The inhibition of protrusional activity, cell spreading, and cytokinesis induced by fumonisin B1 can be reversed by ganglioside GM3. J. Biol. Chem. *272*, 1558–1564.

Moeendarbary, E., Valon, L., Fritzsche, M., Harris, A.R., Moulding, D.A., Thrasher, A.J., Stride, E., Mahadevan, L., and Charras, G.T. (2013). The cytoplasm of living cells behaves as a poroelastic material. Nat. Mater. *12*, 253–261.

Montagnac, G., Echard, A., and Chavrier, P. (2008). Endocytic traffic in animal cell cytokinesis. Curr. Opin. Cell Biol. *20*, 454–461.

Mullen, T.D., Spassieva, S., Jenkins, R.W., Kitatani, K., Bielawski, J., Hannun, Y.A., and Obeid, L.M. (2011). Selective knockdown of ceramide synthases reveals complex interregulation of sphingolipid metabolism. J. Lipid Res. *52*, 68–77.

Mullins, J.M., and McIntosh, J.R. (1982). Isolation and initial characterization of the mammalian midbody. J. Cell Biol. *94*, 654–661.

Murthy, K., and Wadsworth, P. (2005). Myosin-II-dependent localization and dynamics of F-actin during cytokinesis. Curr. Biol. *15*, 724–731.

Ng, M.M., Chang, F., and Burgess, D.R. (2005). Movement of membrane domains and requirement of membrane signaling molecules for cytokinesis. Dev. Cell *9*, 781–790.

Pietuch, A., and Janshoff, A. (2013). Mechanics of spreading cells probed by atomic force microscopy. Open Biol. *3*, 130084.

Saario, S.M., McKinney, M.K., Speers, A.E., Wang, C., and Cravatt, B.F. (2012). Clickable, photoreactive inhibitors to probe the active site microenvironment of fatty acid amide hydrolase(). Chem. Sci. *3*, 77–83.

Sackmann, E. (1996). Supported membranes: scientific and practical applications. Science *271*, 43–48.

Saghatelian, A., Trauger, S.A., Want, E.J., Hawkins, E.G., Siuzdak, G., and Cravatt, B.F. (2004). Assignment of endogenous substrates to enzymes by global metabolite profiling. Biochemistry *43*, 14332–14339.

Sagona, A.P., Nezis, I.P., Pedersen, N.M., Liestøl, K., Poulton, J., Rusten, T.E., Skotheim, R.I., Raiborg, C., and Stenmark, H. (2010). PtdIns(3)P controls cytokinesis through KIF13A-mediated recruitment of FYVE-CENT to the midbody. Nat. Cell Biol. *12*, 362–371.

Schultz, C. (2010). Challenges in studying phospholipid signaling. Nat. Chem. Biol. *6*, 473–475.

Sedzinski, J., Biro, M., Oswald, A., Tinevez, J.Y., Salbreux, G., and Paluch, E. (2011). Polar actomyosin contractility destabilizes the position of the cytokinetic furrow. Nature *476*, 462–466.

Skop, A.R., Liu, H., Yates, J., 3rd, Meyer, B.J., and Heald, R. (2004). Dissection of the mammalian midbody proteome reveals conserved cytokinesis mechanisms. Science *305*, 61–66.

Stewart, M.P., Toyoda, Y., Hyman, A.A., and Müller, D.J. (2012). Tracking mechanics and volume of globular cells with atomic force microscopy using a constant-height clamp. Nat. Protoc. *7*, 143–154.

Stumpf, C.R., Moreno, M.V., Olshen, A.B., Taylor, B.S., and Ruggero, D. (2013). The translational landscape of the Mammalian cell cycle. Mol. Cell *52*, 574–582.

Sullan, R.M., Li, J.K., and Zou, S. (2009a). Direct correlation of structures and nanomechanical properties of multicomponent lipid bilayers. Langmuir *25*, 7471–7477.

Sullan, R.M., Li, J.K., and Zou, S. (2009b). Quantification of the nanomechanical stability of ceramide-enriched domains. Langmuir *25*, 12874–12877.

van Meer, G., Voelker, D.R., and Feigenson, G.W. (2008). Membrane lipids: where they are and how they behave. Nat. Rev. Mol. Cell Biol. *9*, 112–124.

Yokokawa, M., Takeyasu, K., and Yoshimura, S.H. (2008). Mechanical properties of plasma membrane and nuclear envelope measured by scanning probe microscope. J. Microsc. *232*, 82–90.

Zhang, X., Wang, W., Bedigian, A.V., Coughlin, M.L., Mitchison, T.J., and Eggert, U.S. (2012). Dopamine receptor D3 regulates endocytic sorting by a Prazosin-sensitive interaction with the coatomer COPI. Proc. Natl. Acad. Sci. USA *109*, 12485–12490.

Supplemental Information

EXTENDED EXPERIMENTAL PROCEDURES

Cell culture media and supplements were purchased from Sigma and Invitrogen. LC-MS solvents were purchased from Honeywell Burdick & Jackson. Anti-α tubulin (DM1α), anti FIP3-RAB11 (HPA028631), TRITC-phalloidin (P1951), Nile Red (N3013) and micrococcal nuclease (N3755) were purchased from Sigma. WGA (W-11261) and FM4-64 (F34653), CTB1-Alexa488 and conjugated secondary antibodies were purchased from Invitrogen. Anti-mouse integrin-β3 (ab7167) and anti-CHMP2B (ab33174) were purchased from Abcam. Anti-Ceramide antibody was purchased from Glycobiotech GmbH (MAB_0010). Anti-mouse IgG coupled magnetic beads (MagCellect Ferrofluid, anti-mouse IgG beads) were purchased from R&D Research. LC-MS columns were purchased from Phenomenex. All lipid standards used were purchased from Avanti Polar Lipids.

Cell Culture

All HeLa cells, including S3 and GTRH (Green-Tubulin Red-Histone stable cell line, a kind gift from Daniel Gerlich (Steigemann et al., 2009)) were grown at 37°C in complete DMEM (DMEM (Invitrogen) supplemented with 10% fetal bovine serum (FBS) (Sigma) and 1% penicillin/streptomycin (Invitrogen)) in 10 cm dishes (Corning). HeLa GTRH medium was supplemented with 500 μg/ml G418.

Force Spectroscopy Measurements on Cells Using High-Indenting Forces

HeLa cells (150,000) were seeded in a 60 mm plastic cell culture dish (Greiner bio-one, Frickenhausen, Germany) 24h prior the AFM experiment. Force Spectroscopy experiments were performed using a commercial AFM (Dimension Icon, Bruker AXS, Karlsruhe, Germany) equipped with a custom-made temperature controller. The controller is composed of a 16x6x0.5 cm aluminum plate with two silicone heating mats located at each of its ends. The dish containing the cells is located at the center of the plate and a type K thermocouple probe (Fluke, WA, USA) is immersed near the edge of the dish making contact with its bottom surface. This design has a temperature resolution of ± 0.1°C and a set point stability of ± 1°C.

Prior to experiments, the spring constant of the AFM cantilevers (MLCT tip F, k = 0.6 N/m, Bruker AXS, Karlsruhe, Germany) was individually calibrated against a freshly cleaved mica surface in MilliQ water. Then, the heater was turned on, let stabilize, and the HeLa cells were transferred to the AFM and let equilibrate for around 30 min. In the absence of CO_2, media was buffered with 50mM HEPES during the experiment. Target cells were identified with the use of the AFM's built-in optical microscope. Only cells that were sitting far apart from other neighboring cells were indented, in order to avoid indenting regions with overlapping cytoplasms from adjacent cells. Dividing cells were identified according to their morphology (e.g., Figure 3A).

Once a target cell was identified, the AFM probe was allowed to indent the entire cell at a constant velocity of 1 μm sec^{-1} until the culture dish substrate was encountered (applying forces in the range of 10-250 nN), and the probe was subsequently retracted. The resulting force-extension trace features a steady increase in force, as a result of the complex combined effect of deforming the cytoplasm (consisting of a porous elastic solid meshwork (cytoskeleton, organelles, macromolecules) bathed in an interstitial fluid (cytosol)) (Moeendarbary et al., 2013). The complexity of these force-extension traces, where a variety of cellular structures are plastically deformed, precludes the fitting of purely elastic deformation models to the entire force-indentation curve. Once the cell has been completely deformed, and just before the AFM cantilever tip reaches the hard substrate, a discontinuity (or rip) in the force versus extension trace can be easily identified (Yokokawa et al., 2008). Each discontinuity corresponds to the breakthrough of an individual lipid bilayer, featuring an average thickness of 2.5 ± 0.9 nm (n = 704). Crucially, the number of 'jumps' or discontinuities varies according to the cell region. These observations concur with the results previously reported (Yokokawa et al., 2008), where it was clearly demonstrated that the 'rips' or discontinuities observed in the indentation curves on live cells just before contacting the hard substrate and occurring at high applied forces correspond to the penetration of the AFM probe through the plasma membrane. In the Yokokawa et al. experiments, the number of 'rips' varied with the region of the cell being indented. Pushing through the cell peripheries mainly gave rise to one rip (with a second rip being rarely observed), raising the possibility that both the upper and bottom membranes were simultaneously penetrated. In contrast, when indenting the nuclear envelope region, a second rip was observed, which was composed of several small rips, suggesting that the different nuclear membranes were penetrated by the AFM probe. These results were further confirmed by indenting isolated nuclei, revealing multiple rips. In the present study, while indentation on the cytoplasm region clearly reveals two breakthrough events (corresponding to the penetration of the cell's "dorsal" membrane and the substrate-attached "ventral" membrane, respectively), when indenting the cell's nucleus we observe 6 breakthrough events (which correspond to the outer and inner cell membranes (2) plus the indentation of the outer and inner nuclear membrane, each of which is a double bilayer (4)). Altogether, our observations agree with the results reported by Yokokawa et al. in that we observe rips when indenting live cells at higher forces, and also that the number of rips increases when the indentations are carried out on the cell nucleus rather than on the cytoplasm. A key point in our experiments is that each rip or discontinuity is typically formed by a single membrane (confirmed by the length associated with each particular discontinuity). In the experiments reported by Yokokawa et al., many of the observed rips were larger (although the total length of each rip was unfortunately not measured in these experiments), corresponding to the simultaneous breakthrough of a few individual bilayers. Interestingly, in our experiments we show that the thickness of the lipid membranes of live cells closely corresponds to the values measured when such lipid bilayers are supported on a solid substrate (Garcia-Manyes et al., 2005a, 2005b, and 2010) (Figure S2 E-G), thus providing further confidence that each breakthrough event corresponds to the indentation of a single lipid bilayer by the AFM probe. The force at which the tip

penetrates the bilayer, "breakthrough force," is hence the maximum force that the membrane is able to withstand before breaking (plastic deformation), and hence it can be regarded as a true molecular fingerprint (Garcia-Manyes et al., 2010; Garcia-Manyes and Sanz, 2010).

For each individual cell, about 5-6 indentations were performed to avoid the effects of cell damage through consecutive force-extension traces. The breakthrough force of live cell membranes depends on the cell thickness, as reported before elsewhere (Yokokawa et al., 2008). This is probably due to a large contact area between the probe and the cell surface in the case of a deep indentation. Crucially, the breakthrough force also depends on the region of the cell being indented (cytoplasm or nucleus) and the state of division of the cell (dividing or nondividing). Due to the mechanism of cell division, dividing cells are, on average, thicker than nondividing cells, thus leaving a window of cell thickness spanning from 6-7.6 μm (cytoplasm region) (Figure 2) and 4.1-8.3 μm (nucleus) (Figure S2) amenable for comparison. In each case, for comparable cell thickness values, dividing cells are always more mechanically stable (>99.99%, t test) than nondividing cells.

The cantilevers used for these experiments (MLCT tip F, $k = 0.6$ N/m, Bruker AXS, Karlsruhe, Germany) were imaged using SEM (HITACHI S4000, Tokyo, Japan). The Bruker MLCT cantilever has a length of 5.7 μm, and thus somewhat shorter than the thickness of some of the probed cells. Since the shape of the deformed cells as they are being indented by the pyramidal tip is unknown, it is plausible that, at large forces, the contact area between the cell and the cantilever can have contributions both from the pyramidal tip and the cantilever underside (Harris and Charras, 2011). We attempted to address this potential issue by performing the experiments with cantilevers having longer tips. Unfortunately, most of them (e.g., Bruker OLTESPA and FESPA) were found nonsuitable for measurements in liquid environment, due to the inadequacy of their aluminum coating. Therefore, in this work, some caution should be taken with the absolute values in breakthrough force recorded for cells exhibiting very large heights. However, it must be emphasized that this potential bias does not affect our conclusions, which are based on the large differences in the relative (not absolute) values of breakthrough forces obtained for dividing versus nondividing cells that exhibit similar cell heights.

Mitosis and Cytokinesis Arrest

To obtain mitosis and cytokinesis cells, HeLa S3 cells were arrested at prometaphase by nocodazole followed by mitotic shake-off (for mitosis) and release until telophase (for cytokinesis).

Cells were plated in complete DMEM to a final concentration of 4×10^5 cells/mL in 10 cm dishes (4×10^6 cells in 10 mL) and were grown for 24 hr. After 100 nM nocodazole addition for 12 hr, mitotic cells were collected by mitotic shake-off. Plates were washed with cell suspension very gently to collect all mitotic cells. Cell suspensions were collected in Falcon tubes and centrifuged at 200xg and cells were washed with 25 ml PBS/plate. An additional washing step with 10 ml complete DMEM/plate was carried out to remove nocodazole completely. These pellets were used for the lipidomic analysis of mitotic cells. For release into cytokinesis, cells were resuspended in 1.5 ml complete DMEM and plated in 6-well plates (3 ml cell suspension/well). Cells were incubated for 2 hr (cells that were used for midbody isolation were incubated with 2 μM Taxol for 10 min before harvesting). Cells were pelleted by washing the wells with a pipette and spinning down at 200xg. After pelleting this suspension, cells were washed with 4 ml PBS/well. The synchronization efficiency was determined by immunofluorescence. ~80% of the cells were at cytokinesis.

Preparation of Lipid Bilayers for Atomic Force Microscopy

HeLa S3 cell pellets synchronized in S phase or cytokinesis were resuspended in 1 ml PBS. In order to reduce potential protein contamination, the samples were subjected to two cycles of Dounce-homogenization in 1:1:2 PBS:MeOH:$CHCl_3$ and centrifugation at 500xg for 10 min followed by separation of the chloroform layer. This solution was used to prepare small unilamellar vesicles following the method described previously (Garcia-Manyes et al., 2010). Briefly, a 100 μl aliquot of the chloroform solution was evaporated under nitrogen flow for 40 min to ensure the absence of organic solvents. After adding 1 ml of buffer solution (150 mM NaCl, 20 mM $MgCl_2$, 10 mM HEPES pH = 7.4), samples were subjected to 5 cycles of 40 s vortexing at 60°C. The solution was finally sonicated for 30 min and then deposited for 30 min onto freshly cleaved Muscovite Mica (Ted Pella, Redding, CA). Before observation with AFM, the samples were thoroughly rinsed with buffer.

Observation of Lipid Bilayers Using Atomic Force Microscopy

Samples were imaged with a commercial Dimension Icon (Bruker, Karlsruhe, Germany) in tapping mode using V-shaped Si_3N_4 tips (SNL tip A, Bruker, Karlsruhe, Germany) of 0.35 N/m nominal spring constant. Each cantilever was individually calibrated using the equipartition theorem (thermal noise) method before force-distance curves were recorded. Right after imaging a lipid patch, Force-Extension curves were performed to a set of chosen locations at a constant velocity of 1000 nm/s. All experiments were performed at room temperature ranging from 20-24°C. AFM images were analyzed and processed with Nanoscope Software (Bruker, Karlsruhe, Germany). In order to measure the height of each independent phase from the tapping mode images, we flattened each particular image and made a histogram of the height corresponding to all pixels of the image. The height of each individual phase was measured by taking the central value of each distinct peak in the histogram with respect to the mica surface. Force Spectroscopy data were analyzed using a custom-made routine written in Igor Pro Software (Wavemetrics, Portland, OR). The distribution of breakthrough force data shown in Figure 2F corresponds to the sample area shown in Figure 2D and 2E, respectively. The same trend in the mechanical stability of each phase is reproducibly observed when using cantilever tips and samples within independent days of experiment.

Midbody Isolation

The procedure used to isolate midbodies is here described in 3 steps.

Cell Lysis

HeLa S3 cells arrested at cytokinesis were immediately used for midbody isolation. The pellets were resuspended in Mb buffer (20 mM PIPES pH 6.8, 1 μM Taxol supplemented with protease inhibitor cocktail). For each well in the 6 well plates used for cytokinesis release, 0.5 ml Mb buffer was used (~4×10^6 cells with ~80% of the population at cytokinesis). All the steps after this stage were carried out on ice unless specified otherwise. Cell lysis was achieved mechanically with a 27 G 1/2 needle by forcing the cell suspension through the needle 35 times / 1.5 ml of cell suspension. This suspension was further gently vortexed in the presence of 150-212 μm sized glass beads. DNA was digested by adding 0.2 U/μL of micrococcal nuclease. The reaction was started by adding 1 mM $CaCl_2$ and incubated for 10 min at 28°C. The reaction was stopped by the addition of 2 mM EGTA and the suspension was left on ice for 10 min followed by rotation at 4°C for 30 min.

Short Spins

Supernatants were collected and subjected to the following consecutive short spins: Samples were transferred into 2 ml tubes and first centrifuged at 30xg for 7 min. Supernatants were removed and recentrifuged at 70xg for 7 min. At this stage, midbodies were observed in the supernatant of the resulting solution. The remaining supernatants were collected and midbodies were pelleted at 1000xg. The resulting pellets were resuspended in 250 μl Mb buffer.

Immunoprecipitation to Remove Nonmidbody Impurities in Midbody-Enriched Solutions

In a final step, integrin β3 antibody was used to remove nonmidbody impurities. This antibody preferentially binds to membrane structures that co-purified but are not midbodies, allowing further enrichment of midbodies. Anti-mouse IgG coupled magnetic beads from R&D Research were used (MagCellect Ferrofluid, anti-mouse IgG beads). 300 μl beads were washed twice with 500 μl Mb buffer (without Taxol). The beads were resuspended in 150 μl buffer and 150 μl (~10 μg) anti-mouse Integrin β3 antibody was added. The beads were incubated with antibody at room temperature for 1 hr and the beads were washed twice with 500 μl Mb buffer (without Taxol) to remove unbound antibody. This solution was mixed with 250 μl midbody-enriched suspension from the previous step. The mixture was rotated first at room temperature (25 min) then at 4°C (25 min). The supernatant was collected and the beads were gently washed with Mb buffer. The supernatant was combined with the washing solution (final solution) and the beads were discarded. The final solution was pelleted by centrifuging at 1000xg and the pellet was stored at −80°C until further use. The final solution was enriched in midbody structures according to immunofluorescence and electron microscopy studies and used for midbody lipid profiling.

Electron Microscopy

150 μl of midbody suspension was pipetted into 1 ml of 25 mM lysine and 1.5% gluteraldehyde fix in BRB80 in a 5 ml snap cap tube and gently mixed by inverting. Fix for 5 min. The sample and fix were then layered on top of 4 ml of a 30% glycerol BRB80 cushion in a spin down tube with an Aclar coverslip supported by a polycarbonate chock and centrifuged at 5500 rpm for 20 min at 18°C in a JS 13.1 rotor in a Beckman Avanti J-E centrifuge. The cushion and fix were aspirated, the samples rinsed and the coverslip removed, and fixed for 10 min with 1.5% gluteraldehyde in BRB80. They were rinsed with BRB80, then 3X with 0.05M cacodylate buffer pH 7.0. Then the coverslips were postfixed in 1% osmium in 0.8% $K_3Fe(CN)_6$ in cacodylate buffer for 15 min on ice, in hood, followed by rinsing three times in cacodylate buffer and twice in distilled water. Afterward, samples were stained in 1% aqueous uranyl acetate overnight in the dark. They were rinsed in distilled water and dehydrated in an ethanol series using the progressive lowering of temperature method. Coverslips were placed in fresh 100% ethanol and fresh 100% propylene oxide prior to infiltration with solutions of 2:1, 1:2 propylene oxide: epon araldite for 30 min each, then 100% epon araldite for an hour. Samples were mounted and polymerized at 65°C for 48 hr. Areas of midbodies were excised and remounted, serial sectioned at 75 nm on a Leica Ultracut S microtome. Samples were viewed on a Tecnai G2 Spirit BioTWIN and imaged with an AMT 2k CCD camera.

RNAi Screen

We designed a custom RNAi library targeting 244 lipid biosynthetic enzymes purchased from Dharmacon (siGENOME pools). The full list is reported in Table S2. In addition, every plate contained a negative nontargeting siRNA control and siRNA targeting RACGAP1 as positive control.

1.2 μl of 1 μM siRNAs diluted in Dharmacon RNAi buffer and arrayed in 384 well plates was added to clear-bottom 384-well plates (Costar). The siRNAs were incubated for 20 min with 9.4 μl of Interferin transfection reagent diluted to 1/200 in OptiMEM. Approximately 12600 or 6100 HeLa cells/mL (respectively for 72 and 96 hr of incubation time) in DMEM with 10% FBS were added and then the plates were centrifuged at 500 rpm for 1 min. Final volume was 40 μl per well and the final siRNA concentration 30 nM. One day after transfection, 20 μl of DMEM with 10% FBS and 3% Penicillin/Streptomycin per well were added. Plates were fixed respectively 48 or 72 hr later in PBS with 2% Paraformaldehyde, 0.1% Triton X-100 incubated for 20 min. Plates were then incubated with blocking buffer (PBS, with 1% BSA and 10 mM Glycine) for 20 min and stained overnight at +4°C with the primary antibody anti-tubulin diluted in PBS, with 1% BSA (washing buffer). Plates were then washed and stained with DAPI (1.5 μg/ml), Phalloidin-TRITC (8 μg/ml) and secondary antibody-FITC (6.5 μg/ml) in the washing buffer for 1 hr at room temperature. Plates were then rinsed with the washing buffer and imaged using the macro plate reader on a Ti Eclipse Nikon epifluorescence microscope.

The RNAi screen was performed twice with two different incubation times: 72 and 96 hr.

Hits with a percentage of binucleated cells above 10% were considered positive and only those hits that repeated in at least 3/4 experiments (for hits positive after 72 hr) and 2/2 experiments (for the 96 hr hits) were selected. The average percentage of multinucleated cells was calculated using a minimum of 100 cells per case. Hits were then divided into strong > 20% binucleated cells; medium < 20% and > 14% binucleated cells and low < 14% and > 10% binucleated cells.

Individual siRNA were tested for SMPD4 (3/4 positive), GALC (3/4 positive) and DGAT2 (2/4 positive).

Follow-up experiments for time-lapse movies and LC-MS were carried out at 60 nM using a pool of the three positive individuals for SMPD4 (UGAAUCCGUUCGAGUAUUA-GGCCAGGACUGCAAGUACA-AGGUGAAGAGCCACGUCUA) and GALC (CUGG CAACGCCGAGCGAAA-GAGAAUUAUUUCCGAGGAU-GAAAGGAGGAAGCUACGUA) and the siGENOME pool for DGAT2.

Time-Lapse Acquisition

For time-lapse movies of siRNA treated cells, cells were prepared similarly as for the screen, but grown in Ibidi μ-slides 8 wells and imaged after 36 hr (siDGAT2) or 72 hr (siGALC and siSMPD4). Movies were recorded overnight on a Nikon Ti inverted microscope at 37°C and 5%CO_2 in complete DMEM supplemented with 10 mM HEPES at one image/5 min rate.

Immunofluorescence for Cells

HeLa cells were grown on glass coverslips in 24 well plates for 24 hr. Samples were fixed with 2% Paraformaldehyde for 20 min, washed and permeabilized with 0.5% Triton X-100 for 5 min. Blocking and staining was performed as described for the screen. The coverslips were then transferred to an object slide with a drop of ProLong Gold antifade reagent for mounting.

Image Acquisition and Processing of Cells

Images were recorded on a Nikon Ti inverted microscope using either a 20x air or a 60x oil objective. When specified, images were acquired using a 100x oil objective either on a confocal microscope Nikon A1 spectral or on a N-SIM super-resolution microscope at the Nikon Imaging Centre at King's College London. Both the confocal and the N-SIM microscopes were equipped with a 488nm laser for FITC excitation and respectively with a 568nm and 561nm laser for TRITC excitation. Original data were processed using NIS Elements software, ImageJ and Adobe Photoshop® CS3.

Immunofluorescence for Midbody Preparations

Midbody suspensions were placed on coverslips and allowed to settle. The buffer was removed and the structures were fixed with ice-chilled methanol for 5 min, 3.7% formaldehyde in 1x PBS, 1x BRB80 (80 mM PIPES pH 6.8, 1 mM $MgCl_2$, 1 mM EGTA) or 1x HBSS (Hank's buffered salt solution) for 20 min. The coverslips were washed with 1x TBS-Tx (Tris buffered saline with 0.1% Triton X-100), 1x PBS or 1x HBSS and blocked for 30 min at room temperature or at 4°C overnight. After transferring the coverslips to dark chambers, the coverslips were washed with the according buffers and incubated with the primary antibody or dye for 20 min to 2 hr. The coverslips were then washed with the according buffer and incubated with the secondary antibody for one hour when applicable at room temperature. The coverslips were again washed and transferred to an object slide with a drop of ProLong Gold antifade reagent for mounting.

Image Acquisition and Processing of Midbody Preparations

All images were recorded on a Nikon Ti inverted microscope equipped with Perfect Focus System Yokagawa CSU-10 spinning disk confocal unit with 488 nm, 568 nm and 647 nm laser lines at the Nikon Imaging Center at Harvard Medical School. Original confocal data were processed using MetaMorph image acquisition software and Adobe Photoshop CS3.

Force Spectroscopy Measurements on Cells at Low-Indenting Forces; Measurement of Cell Elasticity

siRNA treated cells were prepared similarly as described in the RNAi screen section and grown in 60 mm culture dishes. Cells were maintained at controlled temperature and in buffered medium during the measurements as described here in the previous AFM section. In order to test the elasticity (Young's modulus) of SMPD4 siRNA treated cells as opposed to cells treated with control siRNA, we conducted force-volume experiments whereby the cell surface was regularly indented at 4 μm sec^{-1} applying forces lower than 1.5 nN in the X and Y directions in maps of 16x16 or 32x32 points. In this study, the scan size was set, for most cases, in the range 10-30 μm, depending on the size and shape of the cell cytoplasm, with a resolution between 0.3-2 μm/pixels. For these studies, a cantilever with a lower spring constant (Bruker MLCT, tip C, $k = 0.01$ N/m, Bruker AXS, Karlsruhe, Germany) was used. We developed our own computational tools, written in Igor Pro (Wavemetrics, Portland, OR), to extract the sample stiffness from the raw force-volume files. In this analysis, once the contact point between the tip and the sample is detected by finding the intersection point that best fits the change in trend between a linear and a quadratic function, the stiffness of the contacting region is computed by using the Hertz model for a pyramidal geometry, which allows to calculate the Young's modulus of the sample cell, E, from the evolution of the force, F, as a function of the indentation depth, δ, according to the equation (Harris and Charras, 2011):

$$F = \frac{1.4096 \cdot E \cdot \tan\varphi}{2 \cdot (1 - \upsilon^2)} \cdot \delta^2$$

where υ is the Poisson ratio and φ, the opening angle of the pyramid. We imaged by SEM the tip used during the experiments (Bruker MLCT tip C, Bruker AXS, Karlsruhe, Germany), and measured the tip height of 5.74 μm, $\varphi = 29.8^\circ$ and $R = 50$ nm. Assuming a $\upsilon = 0.5$, the value of the Young's Modulus was obtained by fitting the Hertz equation to each of the 4314 (siSMPD4 cells) and 4098 (control cells) individual force-extension traces. The Hertz model is derived for an infinitely thick sample. Hence, data should be fitted if the indentation is small compared with the thickness of the sample. Each trace was fitted within the force range below 600 pN, which corresponds to a tip indentation of 500 nm at most, which is small compared to the average height of dividing cells, about 5-10 μm. (Kihara et al., 2013) Therefore, the *E* values obtained were not significantly affected by the underlying stiff substrate. Each individual trace was manually inspected to ensure that no plastic deformation of any sort (discontinuity in the force-distance trace) occurred within the fitted force range. Each indentation force-volume map was individually inspected in order to discard any possible outliers that might mistakenly appear in the rims of the cell and corresponding instead to the substrate.

Individual Force Volumes presented local differences of stiffness depending on the sampled region of the cytoplasm. In any case, these differences are rather small compared to the cell to cell differences in mechanical properties. Each force volume can be defined by a single stiffness histogram. Each distinct histogram shows a unimodal distribution of stiffness values with a positive skew. The overall histogram that includes the results of all individual force volume data sets shows a distribution of stiffness values that cannot be accounted for variability within experimental days or used AFM probes.

The values of Young's modulus that we obtained (kPa for both dividing and nondividing cells) are similar to those observed by others for a variety of different cells, including HeLa cells (Yokokawa et al., 2008), and ranging between ~1-30 kPa.

LC-MS Analysis

Preparation of Lipid Extracts for LC-MS

Cell pellets were resuspended in 1 ml PBS and Dounce-homogenized in 1:1:2 PBS:MeOH:$CHCl_3$ (Saghatelian et al., 2004). The resulting suspension was centrifuged at 500xg for 10 min followed by the separation of the chloroform layer from the aqueous layer. In order to increase the concentration of lipids, samples were evaporated and redissolved in 100 μl chloroform.

LC-MS analysis was performed using an Agilent 6520 Series Accurate-Mass Quadrupole Time-of-Flight LC-MS (supported by the Taplin Funds for Discovery Program at Harvard Medical School (P.I., Suzanne Walker)). For LC-MS analysis in negative mode, a Gemini C18 reversed phase column (50 μm, 4.6 mm x 50 mm) from Phenomenex was used together with a reversed phase guard cartridge (C18, Phenomenex). In positive mode, a Luna C5 reversed phase column (50 μm, 4.6 mm x 50 mm) from Phenomenex was used together with a reversed phase guard cartridge (C5, Phenomenex). Stationary phase A was a 95:5 H_2O:MeOH, and mobile phase B was 60:35:5 2-propanol:MeOH:H_2O. 0.1% formic acid and 5 mM ammonium formate for the positive ionization mode, and 0.1% ammonium hydroxide for the negative ionization mode, were added prior to LC-MS runs.

Flow rate started at 0.1 mL/min for 5 min followed by a flow rate of 0.5 ml/min for the duration of the gradient. The gradient started after 5 min at 0% B and then increased to 100% B over the course of 70 min followed by an isocratic gradient of 100% B for 8 min before equilibration for 7 min at 0% B. The total analysis time was 90 min. MS analysis was performed using an Agilent ESI-TOF fitted electrospray ionization (ESI) source. The capillary voltage was set to 3500 V and the fragmentor voltage to 175 V. The drying gas temperature was 350°C, the drying gas flow rate was 12 L/min and the nebulizer pressure was 30 psi. Untargeted data were collected using a mass range of 100-1700 Da in extended dynamic range mode, and each run was performed using 30 μl injections of lipid extract. In each run, we analyzed alternating treated and control samples (three each). Multiple (at least three) independent experiments were used for final profiling analysis. MS/MS experiments were carried out in a similar fashion, but different collusion energies were tested to find the optimal ionization. Fragmentation patterns were observed at 15 V, 35 V, 55 V and 75 V.

Data Analysis

For lipid profiling, analysis of the resulting total ion chromatograms was performed using the software package XCMS (http://metlin.scripps.edu/xcms) (Ivanova et al., 2004; Smith et al., 2006). Files were converted from Agilent Masshunter format (.d) to .mzXML files with the software "trapper." Treated and untreated samples were compared and ranked according to fold change, peak size and statistical significance.

Lipidomic Analysis of Cytokinesis versus S Phase Cells and the Midbody Region

Cytokinesis and S phase cells were obtained as described in the main text and above. The samples were normalized based on protein concentration before lipid extraction using a Bradford Assay. LC-MS analysis was carried out as described above. Three independent runs were used for negative and positive modes. For S phase samples, on average, 1/3 of a confluent 10 cm flask of S phase cells was used for each LC-MS sample (each profiling used three samples). For cytokinesis samples, on average, 1/4 of a 6-well plate was used for each LC-MS sample (each profiling used three samples).

To eliminate signals due to lipids from membrane impurities in the midbody, we performed an additional set of profiling experiments where asynchronous cells were subjected to the same midbody purification procedure as cytokinesis cells. We called this "purified lysate" (See Figure 5D).

Midbody enriched samples, purified lysate and cytokinesis cells were normalized based on protein concentration. LC-MS analysis was carried out as described above. Three independent runs each were used for negative and positive modes.

We carried out several steps to determine which m/z species were significantly changed during cytokinesis relative to S phase. First, m/z species obtained from XCMS for each run were sorted based on fold change and p value. m/z species with < 3-fold change and inconsistent isotopic distribution were eliminated. We grouped the peaks based on their isotopic distribution because any

significant m/z peak should be present with peaks corresponding to other isotopes. Next, we manually investigated the peaks in Agilent Qualitative Analysis software. Well resolved and clean peaks were reintegrated and fold changes were recalculated manually. Once accurate fold changes were calculated based on manual integration, we eliminated species with < 3-fold change. Finally, we compared the presence of m/z species in different runs and only considered species that were observed in three independent experiments so that we would only carry on the most reproducible m/z species to the validation step.

Lipidomic Analysis of Cytokinesis versus Mitosis Cells

Cytokinesis and mitotic cells were obtained as described above. The samples were normalized based on protein concentration before lipid extraction using a Bradford Assay. For cytokinesis samples, on average, 1/4 of a 6-well plate was used for each LC-MS sample (each profiling used three samples). The corresponding mitotic cells were prepared accordingly. The experimental set-up and LC-MS analysis were as described above for cytokinesis versus S phase cells.

Lipidomic Analysis of siSMPD4-, siGALC-, and siDGAT2-Treated Cells

Samples were prepared and LC-MS runs were performed as described above except a shorter gradient was used for all runs (the gradient started after 5 min at 0% B and then increased to 100% B over the course of 60 min). Fold increases in specific lipids were calculated by manual integration of the corresponding peaks in siSMPD4, siGALC, siDGAT2 and control siRNA treated cells by using targeted analysis of the lipid species. Experiments were performed in duplicate.

Identification and Validation of Lipids

An initial search based on high resolution molecular weight was performed in the Metlin database to determine potential candidate lipids for the corresponding species (http://metlin.scripps.edu/metabo_search_alt2.php) (Smith et al., 2005). Known lipid standards (or in some cases similar lipid standards due to commercial availability) were purchased for the candidate lipids and MS/MS fragmentation patterns of the species of interest and known candidate lipids were compared. In addition, searches based on MS/MS fragments provided in Metlin were used complementary to the fragmentation pattern of known standards (http://metlin.scripps.edu/fragment_search.php).

Identification and Validation of Ceramides

In order to confirm the nature of lipids that were predicted to be ceramides, we used the fragmentation patterns of C24 ceramide as a standard. C24 ceramide was purchased from Avanti Polar Lipids. C24 ceramide (m/z = 648.6291) ($(M-H)^-$): Following fragments were observed: 237.2228, 349.3468, 366.3722, 392.3873 and 408.3829.

Identification and Validation of m/z = 648.6366

Fragmentation patterns of m/z = 648.6366 were compared to the fragmentation patterns of C24 ceramide (listed above). Following fragments were observed for m/z = 648.6366: 237.2209, 349.3458, 366.3744, 392.3892 and 408.3833. According to this fragmentation pattern, m/z = 648.6366, $(M-H)^-$, was identified as C24 ceramide.

Identification and Validation of m/z = 620.5987

Fragmentation patterns of m/z = 620.5987 were compared to the fragmentation pattern of C24 ceramide (listed above). Following fragments were observed for m/z = 237.2209, 339.3309, 364.3585 and 408.3833. According to this fragmentation pattern, m/z = 620.5987, $(M-H)^-$, was identified as C22 ceramide.

Identification and Validation of diH-Ceramides

In order to confirm the nature of lipids that were predicted to be diH-ceramide, we used the fragmentation patterns of C24 and C16 diH-ceramide as a standards (purchased from Avanti Polar Lipids).

For C24 diH-ceramide (m/z = 650.6226) ($(M-H)^-$) the following fragments were observed: 239.2366, 349.3648 and 376.3596.

For C16 diH-ceramide (m/z = 538.5176) ($(M-H)^-$) the following fragments were observed: 239.2316, 253.2521, 280.2629, and 490.4973.

Identification and Validation of m/z = 538.5216

Fragmentation patterns of m/z = 538.5216 were compared to the fragmentation patterns of C16 diH-ceramide (listed above). Following fragments were observed for m/z = 538.5216: 239.2244, 253.2254, 280.2630, 490.4947. According to this fragmentation pattern, m/z = 538.5216, $(M-H)^-$, was identified as C16 diH-ceramide.

Identification and Validation of m/z = 566.5518

Fragmentation patterns of m/z = 566.5518 were compared to the fragmentation patterns of C24 diH-ceramide (listed above). For m/z = 566.5518, 239.2367, 283.26385, and 321.3157 fragments were observed. According to this fragmentation pattern, m/z = 566.5518, $(M-H)^-$, was identified as C18 diH-ceramide.

Identification and Validation of m/z = 594.5830

Fragmentation patterns of m/z = 594.5830 were compared to the fragmentation patterns of C24 diH-ceramide (listed above). For m/z = 594.5830, 239.2372 293.2812 and 363.3238 fragments were observed. According to this fragmentation pattern, m/z = 594.5830, $(M-H)^-$, was identified as C20 diH-ceramide.

Identification and Validation of m/z = 622.6170

Fragmentation patterns of m/z = 622.6170 were compared to the fragmentation patterns of C24 diH-ceramide (listed above). Following fragments were observed for m/z = 622.6170: 239.2377, 321.3157 and 339.3309. According to this fragmentation pattern, m/z = 622.6170, $(M-H)^-$, was identified as C22 diH-ceramide.

Identification and Validation of m/z = 652.6599

Fragmentation pattern of m/z = 652.6599 was compared to the fragmentation pattern of C24 diH-ceramide (listed above). Following fragments were observed for m/z = 634.6483: 302.3059, 284.2944, 266.2845 and 254.2844. According to this fragmentation pattern, m/z = 652.6599, $(M+H)^+$, was identified as C24 diH-ceramide.

Identification and Validation of Hexosylceramides

In order to confirm the nature of lipids that were predicted to be hexosylceramides, we used the fragmentation patterns of C16 and C24:1 glucosylceramide as standards (purchased from Avanti Polar Lipids).

For C16 glucosylceramide (m/z = 698.5610) ($(M-H)^-$) the following fragments were observed: 143.0332, 237.2193, 280.2632, 536.5020.

For C24:1 glucosylceramide (m/z = 808.6670) ($(M-H)^-$) the following fragments were observed: 101.0247, 119.0312, 237.2216, 365.3401, 391.3733 and 647.607.

Identification and Validation of m/z = 698.5610

Fragmentation patterns of m/z = 698.5610 were compared to the fragmentation patterns of C16 glucosylceramide (listed above). Following fragments were observed for m/z = 698.5610: 143.0342, 237.2197, 280.2668, and 536.5056. According to this fragmentation pattern, m/z = 698.5610, $(M-H)^-$, was identified as C16 hexosylceramide.

Identification and Validation of m/z = 810.6783

Fragmentation patterns of m/z = 810.6783 were compared to the fragmentation patterns of C24:1 glucosylceramide (listed above). Following fragments were observed for m/z = 810.6783: 101.0219, 119.0392, 237.2226, 367.3596, 391.3645 and 649.6273. According to this fragmentation pattern, m/z = 810.6783, $(M-H)^-$, was identified as C24 hexosylceramide.

Identification and Validation of m/z = 661.5191

Fragmentation patterns of m/z = 661.5191 were compared to fragmentation patterns of phosphatidic acid (16:0/18:1) (m/z = 673.4811) (purchased from Avanti Polar Lipids). Following fragments were observed for m/z = 661.5191 and PA (16:0/18:1), respectively: 255.2387, 423.2947 and 391.2200, 255.2392, 152.9949. According to this fragmentation pattern, m/z = 661.5191, $(M-H)^-$, was identified as phosphatidic acid ether/ester (O-18:0/16:0).

Identification and Validation of m/z = 647.4645

Fragmentation patterns of m/z = 647.4645 were compared to fragmentation patterns of phosphatidic acid (17:0) (m/z = 675.4968) and phosphatidic acid (14:0) (m/z = 591.4028) (purchased from Avanti Polar Lipids). Following fragments were observed for phosphatidic acid (17:0) (m/z = 675.4968) and phosphatidic acid (14:0) (m/z = 591.4028) respectively: 423.2512, 405.2385, 269.2474, 152.9943 and 381.2032, 363.1824, 227.2016, 152.9948. For m/z = 647.4645, 409.2347, 255.2389 and 152.9957 fragments were observed. According to this fragmentation patterns, m/z = 647.4645, $(M-H)^-$, was identified as phosphatidic acid (16:0).

Identification and Validation of m/z = 810.5278

Fragmentation patterns of m/z = 810.5278 were compared to fragmentation patterns of phosphatidylserine (17:0) (m/z = 762.5288) (purchased from Avanti Polar Lipids). Following fragments were observed for m/z = 810.5278 and PS (17:0), respectively: 437.2675, 419.2546, 303.2335, 283.2642, 152.9957 and 405.2393, 269.2470, 152.9950. According to this fragmentation patterns, m/z = 810.5278, $(M-H)^-$, was identified as phosphatidylserine (18:0/20:4).

Identification and Validation of m/z = 792.7113

Fragmentation patterns of m/z = 792.7113 were compared to fragmentation patterns of triacylglycerol (18:2) (m/z = 896.7712) and triacylglycerol (16:0/18:1/16:0) (m/z = 850.7868) (purchased from Avanti Polar Lipids). Following fragments were observed for m/z = 792.7113: 575.5047, 265.2510, 237.2204, 183.1751, 163.1507, 149.1335, 135.1156, 121.1001, 109.1008. (See the diagram below for the detailed assignment of different fragments). 575.5047 fragment is consistent with a C34:2 species. This fragment shows a neutral loss of a C12:0 side chain. The cationic form of the C12:0 fragment is also observed as m/z = 183.1751 (m/z = 183.1751 is a

minor fragment and corresponding its isotopic pattern is not clean due to the presence of other low m/z species which could either be background ions or fragmentation products of other larger species). According to this fragmentation patterns, m/z = 792.7113, $(M+NH_4)^+$, was identified as triacylglycerol (16:1, 12:0, 18:1) (Figure S4).

Identification and Validation of m/z = 837.5525

Fragmentation patterns of m/z = 837.5525 were compared to fragmentation patterns of phosphatidylinositol (16:0) (m/z = 808.5105) (purchased from Avanti Polar Lipids). Following fragments were observed for m/z = 837.5525 and phosphatidylinositol (16:0), respectively: 419.3051, 255.2317, 241.0126, 222.9989, 152.9978 and 391.2263, 255.2324, 152.9949. According to this fragmentation patterns, m/z = 837.5525, $(M-H)^-$, was identified as phosphatidylinositol (16:0/18:0).

Identification and Validation of m/z = 441.3344

A search based on high-resolution molecular weight in the Metlin database predicted that this ion is a sterol derivative. MS/MS experiments yielded the following fragments for m/z = 441.3344: 423.3229, 401.2849, 385.2996, 186.9848 and 149.0014. 423.329 and 401.2849 fragments are $(M+Na-2H-H_2O)^-$ and $(M-H-H_2O)^-$ fragments, respectively. Comparison of these fragments with known fragments in Metlin database suggested that this species could be a trihydroxycholestane derivative. Accordingly, we purchased hydroxyl cholesterol, cholestane and cholestene derivatives (27-hydroxycholesterol, 3β-5α-6β-trihydroxylcholestane and cholest-5-ene-3β, 7β, 25-triol) and compared the fragmentation patterns of these standards with m/z = 441.3344 (standards were purchased from Avanti Polar Lipids). One of the major fragments 385.2996 is predicted to be a 25-hydroxy cholesterol fragment by Metlin and this fragment was also observed in cholest-5-ene-3β, 7β, 25-triol consistent with a hydroxyl group at 25 position. We then compared the fragmentation of cholest-5-ene-3β, 7β, 25-triol to m/z = 441.3344 specifically: 401.2918, 385.2814, 189.8942, 149.0061. According to the fragmentation pattern, m/z = 441.3344, $(M+Na-2H)^-$, was assigned as a trihydroxycholestane where two hydroxyl groups are at the 3- and 25- positions and third hydroxyl group is on the B-ring.

SUPPLEMENTAL REFERENCES

Cases, S., Stone, S.J., Zhou, P., Yen, E., Tow, B., Lardizabal, K.D., Voelker, T., and Farese, R.V., Jr. (2001). Cloning of DGAT2, a second mammalian diacylglycerol acyltransferase, and related family members. J. Biol. Chem. *276*, 38870–38876.

Clarke, C.J., Wu, B.X., and Hannun, Y.A. (2011). The neutral sphingomyelinase family: identifying biochemical connections. Adv. Enzyme Regul. *51*, 51–58.

Garcia-Manyes, S., Oncins, G., and Sanz, F. (2005b). Effect of temperature on the nanomechanics of lipid bilayers studied by force spectroscopy. Biophys. J. *89*, 4261–4274.

Ivanova, P.T., Milne, S.B., Forrester, J.S., and Brown, H.A. (2004). LIPID arrays: new tools in the understanding of membrane dynamics and lipid signaling. Mol. Interv. *4*, 86–96.

Kihara, T., Yoshida, T., Haghparast, S.M.A., and Miyake, J. (2013). Elasticity Mapping Analysis of Apical Cell Periphery Actin Structures of Normal Fibroblasts and Cervical Cancer Cells. JASMI *3*, 124–129.

Lee, W.C., Kang, D., Causevic, E., Herdt, A.R., Eckman, E.A., and Eckman, C.B. (2010). Molecular characterization of mutations that cause globoid cell leukodystrophy and pharmacological rescue using small molecule chemical chaperones. J. Neurosci. *30*, 5489–5497.

Smith, C.A., O'Maille, G., Want, E.J., Qin, C., Trauger, S.A., Brandon, T.R., Custodio, D.E., Abagyan, R., and Siuzdak, G. (2005). METLIN: a metabolite mass spectral database. Ther. Drug Monit. *27*, 747–751.

Smith, C.A., Want, E.J., O'Maille, G., Abagyan, R., and Siuzdak, G. (2006). XCMS: processing mass spectrometry data for metabolite profiling using nonlinear peak alignment, matching, and identification. Anal. Chem. *78*, 779–787.

Steigemann, P., Wurzenberger, C., Schmitz, M.H., Held, M., Guizetti, J., Maar, S., and Gerlich, D.W. (2009). Aurora B-mediated abscission checkpoint protects against tetraploidization. Cell *136*, 473–484.

Wenger, D.A., Rafi, M.A., and Luzi, P. (1997). Molecular genetics of Krabbe disease (globoid cell leukodystrophy): diagnostic and clinical implications. Hum. Mutat. *10*, 268–279.

Wu, B.X., Clarke, C.J., and Hannun, Y.A. (2010). Mammalian neutral sphingomyelinases: regulation and roles in cell signaling responses. Neuromolecular Med. *12*, 320–330.

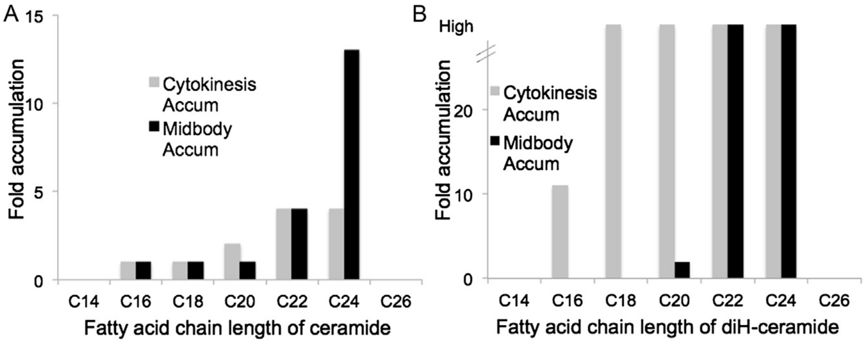

Figure S1. Targeted MS Analysis of the Ceramide and diH-Ceramide Content of Cytokinesis Cells and Midbodies, Related to Figure 1

(A) Fold changes of ceramide species with respect to the fatty acid chain length in cytokinesis cells (gray, compared to S phase cells) and at midbodies (black, compared to purified lysate).

(B) Fold changes of diH-ceramide species with respect to the fatty acid chain length in cytokinesis cells (gray, compared to S phase cells) and at midbodies (black, compared to purified lysate).

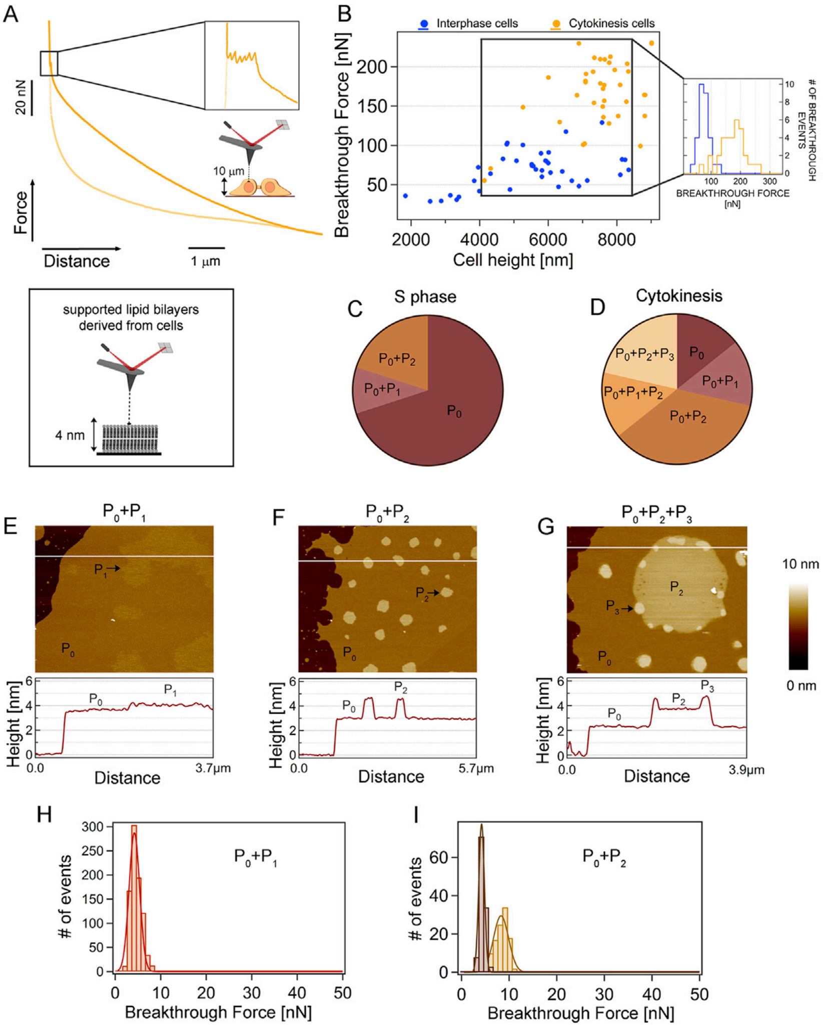

Figure S2. Cytokinesis Cells Exhibit Higher Mechanical Resistance Than Interphase Cells in Distinct Cellular Regions; High-Resolution AFM Images on Supported Lipid Bilayers Reveal a Complex Distribution of Phases, Related to Figure 2

(A) Typical force-distance curve conducted on a live dividing HeLa cell in the nuclear area. This event is distinguishable by six consecutive discontinuities corresponding to the breakthrough of the plasma membrane (two events) in addition to those of the nuclear envelope (composed by two lipid bilayers and therefore showing four breakthrough events).

(B) Scatterplot of the upper membrane breakthrough force (first event) versus the cell height for interphase (blue) and cytokinesis cells (yellow) in the nuclear area (19 cells per case). As in the case of the cytoplasmic region, the breakthrough force steadily increases with the cell height. When selecting a comparable range of cell heights (4.1-8.3 µm), the mechanical resistance of the nuclear region in cytokinesis membranes is clearly higher (174 ± 42 nN, n = 34) than that of nondividing cells (76 ± 19 nN, n = 29) (inset in B) with a > 99.99% confidence (Student's t test).

(C–I) Supported lipid bilayers isolated from live cells and analyzed at high-spatial resolution by AFM imaging exhibit a complex distribution of phases and mechanical stabilities. While Figure 2 D–E reflects the most distinctive patterns of phase distribution in our supported lipid bilayer samples, in some of the preparations additional phase distributions were observed. (C) Pie chart corresponding to S phase preparations shows that, while samples showing only a P_0 phase are predominant (Figure 2D), in some cases P_0 is accompanied by P_1 or P_2 phases. Note that the nomenclature defining each of the phases is arbitrary, yet self-consistent throughout the paper. (D) In the case of lipid bilayers formed from cytokinesis cells, a wider combination of the four distinct observed phases is encountered, as shown in the pie chart. The very stiff P_3 phase was never observed in lipid bilayers isolated from S phase cells. Each pie chart contains 10 and 14 sets of experiments for S phase and cytokinesis cells, respectively. Particularly interesting phase combinations that are not discussed in the main text for cytokinesis preparations are shown in E and F. (E) A P_1 phase that is only slightly higher than the matrix P_0 phase (<0.2 nm) and readily apparent in the phase channel (not shown). (H) Notably, there is no measurable difference in the mechanical stability of the P_0 and P_1 phases (3.7 ± 1.7 nN). (F) Another common combination of phases is observed in F, whereby only P_0 and P_2 phase coexist. In this case, the height of the P_2 phase is significantly higher (>1.27 nm) than the matrix P_0 phase. (I) The mechanical stability of the P_2 phase is higher (8.0 ± 1.3 nN) than the P_0 phase (3.7 ± 0.6 nN).

A larger version of Figure 2E with the associated height profile is shown in G. In this case three main phases are observed: a first P_0 phase exhibiting 2.86 ± 0.52 nm, a second phase (P_2) showing a height of 4.13 ± 0.54 nm relative to the mica substrate, and a higher P_3 phase, featuring 4.96 ± 0.16 nm. For S phase cells, an average height of 2.63 ± 0.72 nm is observed for the matrix, continuous P_0 phase (ref. to Figure 2D, height profile not shown).

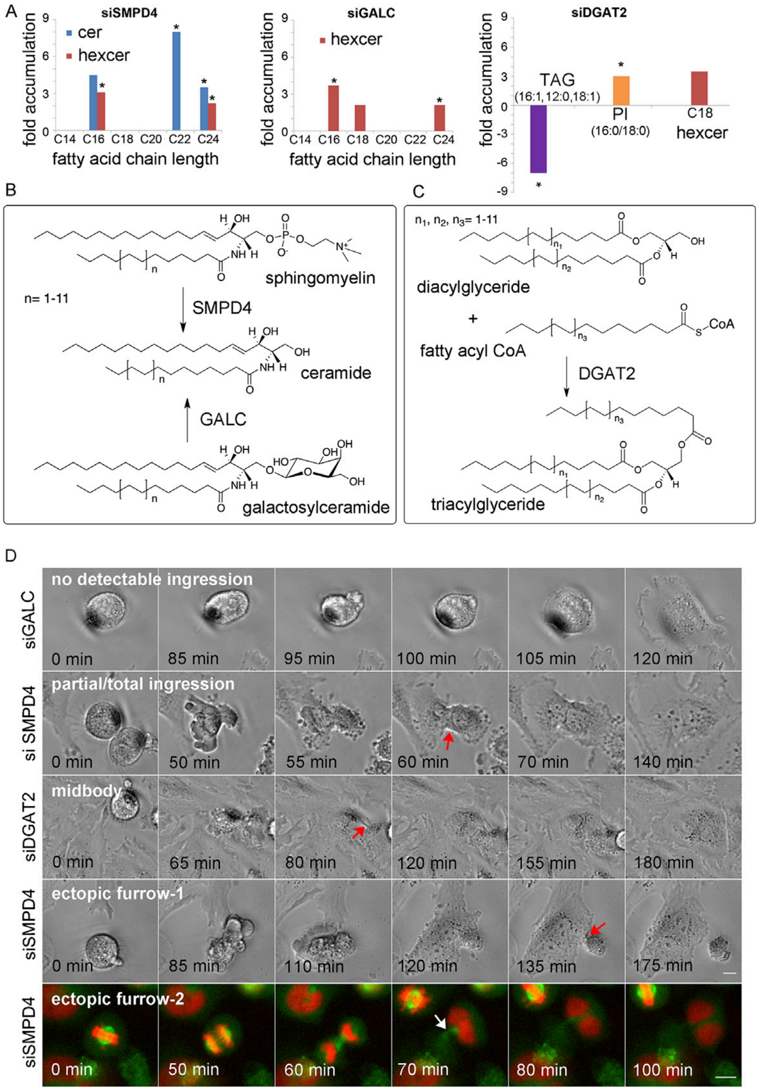

Figure S3. Targeted MS Analysis of Lipids Changed in Cells Treated with SMPD4, GALC, or DGAT2 siRNA, Proposed Reaction Scheme for These Enzymes, and Cell Division Failure Phenotypes for RNAi-Treated Cells, Related to Figure 3

(A) Fold changes in ceramides, diH-ceramides, hexosylceramides, sphingomyelins and the lipids that accumulate during cytokinesis and at the midbody in siSMPD4, siGALC and siDGAT2 treated cells were calculated. For siDGAT2 treated cells, fold changes of representative DAGs (ranging from 24:0 to 38:0 with different unsaturation states) and TAGs (ranging from 36:0 to 50:0 with different unsaturation states) were also calculated. Lipid species that were also accumulated in cytokinesis and/or midbodies are highlighted with an asterisk (*). Ceramides (cer) and hexosylceramides (hexcer) with varying side chains are shown for SMPD4 and GALC. A full list of the targeted analysis of ceramides is shown in Table S3.

(B and C) Proposed reaction schemes for SMPD4, GALC (B) and DGAT2 (C): SMPD4 and GALC catalyze the formation of ceramide from sphingomyelins and galactosylceramides, respectively. DGAT2 catalyzes the formation of triacylglycerides from diacylglycerides and fatty acyl coenzyme A (fatty acyl CoA) (Cases et al., 2001; Clarke et al., 2011; Lee et al., 2010; Wenger et al., 1997; Wu et al., 2010).

(D) Representative still images from time-lapse movies of dividing cells for SMPD4, GALC and DGAT2 siRNA treatments illustrating the different mechanisms that trigger cell division failure in HeLa and HeLa GTRH (Green-Tubulin Red-Histone) cells (last row, ectopic furrow-2). From top to bottom: no detectable ingression (siSMPD4, siGALC and siDGAT2: 7.6%, 14% and 8.3%, respectively); partial/total ingression (= no detectable formation of a midbody: siSMPD4, siGALC and siDGAT2: 38%, 55.5% and 48.6%, respectively); midbody (= clear formation of a midbody, late cytokinesis defect: siSMPD4, siGALC and siDGAT2: 13.9%, 20.8% and 18.1%, respectively); ectopic furrows (= formation of an ectopic furrow and abscission or refusion of a portion of cytoplasm without nucleus: siSMPD4, siGALC and siDGAT2: 40.5%, 9.7% and 25%, respectively). Respective distributions of the different mechanisms for every enzyme knockdown are shown in Figure 3C. Arrows (red and white) highlight the phenotype characterizing each mechanism. Two different examples are shown for the ectopic furrow, they differ in the position and the timing of the ectopic furrow formation and the size of the portion of cytoplasm without nucleus compared to the binucleated cell (grouped in the histogram in Figure 3C). Scale bars, 10 μm.

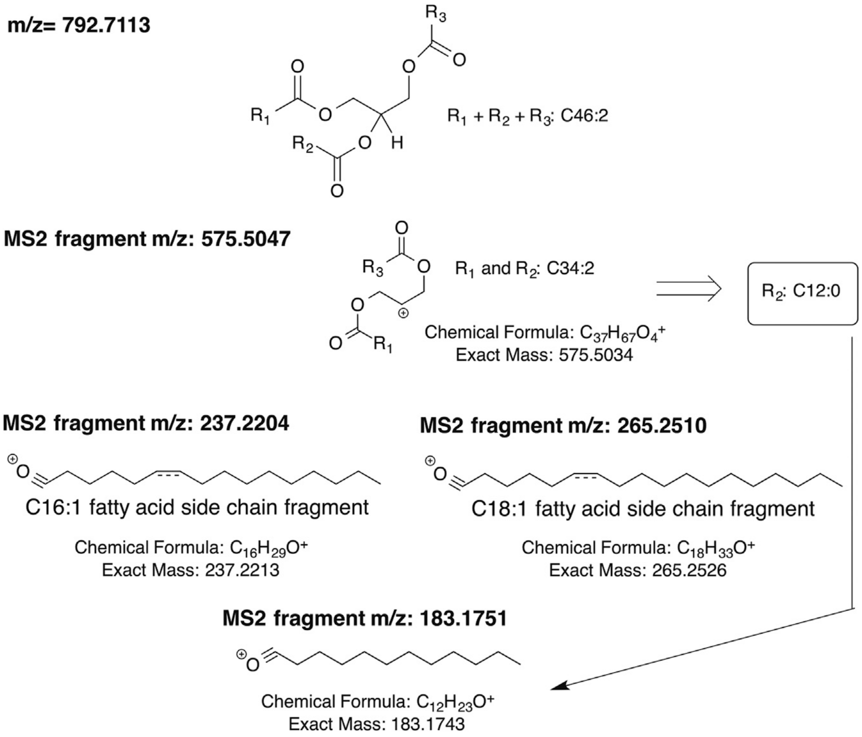

Figure S4. Scheme Showing the Assignment of m/z = 792.7113 as TAG (16:1, 12:0, 18:1), Related to Experimental Procedures
MS/MS spectra for m/z=792.7113 were collected and corresponding fragments were identified. The fragments that were used for characterization are shown above. According to the fragment pattern, m/z=792.7113 was identified as triacylglycerol (16:1, 12:0, 18:1).
